# Efficient routes toward the synthesis of the D-*rhamno*-trisaccharide related to the A-band polysaccharide of *Pseudomonas aeruginosa*

**DOI:** 10.3762/bjoc.10.153

**Published:** 2014-07-01

**Authors:** Aritra Chaudhury, Sajal K Maity, Rina Ghosh

**Affiliations:** 1 Department of Chemistry, Jadavpur University, Kolkata 700 032, India

**Keywords:** A-band polysaccharide, D-*rhamno*-trisaccharide, deoxygenation on thioglycoside, multivalent glycosystems, one-pot sequential glycosylation, *Pseudomonas aeruginosa*

## Abstract

The present work describes efficient avenues for the synthesis of the trisaccharide repeating unit [α-D-Rha*p*-(1→3)-α-D-Rha*p*-(1→3)-α-D-Rha*p*] associated with the A-band polysaccharide of *Pseudomonas aeruginosa.* One of the key steps involved 6-*O*-deoxygenation of either partially or fully acylated 4,6-*O*-benzylidene-1-thiomannopyranoside by radical-mediated redox rearrangement in high yields and regioselectivity. The D-*rhamno-*thioglycosides so obtained allowed efficient access to the trisaccharide target via stepwise glycosylation as well as a one-pot glycosylation protocol. In a different approach, a 4,6-*O*-benzylidene D-*manno-*trisaccharide derivative was synthesized, which upon global 6-*O*-deoxygenation followed by deprotection generated the target D-*rhamno-*trisaccharide. The application of the reported regioselective radical-mediated deoxygenation on 4,6-*O*-benzylidene D-*manno* thioglycoside (hitherto unexplored) has potential for ramification in the field of synthesis of oligosaccharides based on 6-deoxy hexoses.

## Introduction

With the firm establishment of the critical roles played by oligosaccharides in diverse biological processes [1–4], the field of oligosaccharide synthesis has seen a rapid development over the last two decades [5–7]. The D-rhamnoside motif is of particular interest with its presence established in the LPS/EPS systems of various bacterial strains which are pathogenic towards both plants and animals. These species include the *Burkholderia cepacia* complex [8]*, P. aeruginosa* [9]*, Helicobacter pylori* [10], *Citrobacter freundii* [11]*, Campylobacter fetus* [12], *Stenotrophonas maltophilia* [13], *Xanthomonas campestri* [14] and *Brucella sp.* [15]*.*

**Figure 1:**
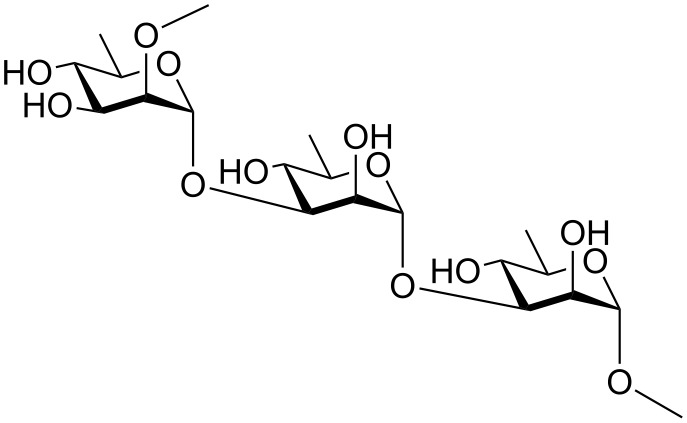
Repeating unit of the A-band polysaccharide of *P. aeruginosa.*

*P. aeruginosa* has long been established as an opportunistic pathogen which infects humans having compromised immunity with fatal consequences in a majority of cases. Its high persistence against a wide variety of antibiotics is also well documented. It has also been observed how the colonized form of this species in cystic fibrosis lungs, through non-expression of *O*-antigens, offer high persistence often rendering *O*-antigen-based vaccines ineffective. But the conservation of the A-band polysaccharide even in the colonized form makes this repeating unit a viable candidate for A-band polysaccharide-based vaccines which can avoid the vulnerability applicable to their *O-*antigen-based counterparts [16]. Hence, the A-band polysaccharide [16] of *P. aeruginosa* which has been characterized previously as a repeating combination of [→2)-α-D-Rha*p*-(1→3)-α-D-Rha*p*-(1→3)-α-D-Rha*p*] (Figure 1) provides for a synthetic target whose efficient synthesis is worth pursuing. It is to be noted that synthesis of a tetrasaccharide, α-D-Rha*p*-(1→2)-α-D-Rha*p*-(1→3)-α-D-Rha*p*-(1→3)-α-D-Rha*p* [17] and a trisaccharide, α-D-Rha*p*-(1→3)-α-D-Rha*p*-(1→2)-α-D-Rha*p* [18] related to the A-band polysaccharide of *P. aeruginosa* were made with a view to develop glycoconjugate vaccines, but none have ultimately materialized into valid vaccine candidates.

Thus, we targeted the trisaccharide [α-D-Rha*p*-(1→3)-α-D-Rha*p*-(1→3)-α-D-Rha*p*] as our synthetic goal toward construction of a probable vaccine candidate against *P. aeruginosa.*

The synthesis of the D-rhamnose-based oligosaccharide from the D-mannose motif has received substantial attention over the last decade. The problem of β-D-rhamnoside synthesis has been greatly addressed by Crich et al. [19–23]. But, Crich’s global deoxygenating strategy, despite its ultimate efficiency, still requires synthetic modification on the conventional 4,6-*O*-benzylidene framework involving reagents which are rather expensive. Moreover, additional steps are required for the preparation of these adequately derivatized mannose-based systems from which the D-rhamnose motif may be accessed. Other reports on the D-rhamnose-based synthesis have also surfaced in recent times [24–25]. Apart from this, the synthesis of monomeric D-rhamnose has been highly streamlined by Roy et al. in 2007 [26], and further improvement on this method was reported by Kiefel et al. in 2011 [27]. However, the deoxygenation protocol involving halogenation under Mitsunobu conditions was found to be inefficient when applied to thioglycosides directly. Moreover, the use of stoichiometric amounts of the toxic tin hydride for radical-based reductive dehalogenation as required by the above method appeared undesirable especially in the preparative stages where reactions have to be set up on a large scale. On the other hand, Kiefel’s method requires four steps for the conversion of D-mannose to D-rhamnose on which further manipulations are required to reach the adequately designed derivatives. This makes the starting material preparations rather long drawn.

Keeping these limitations in mind we selected the protocol devised by Pedersen et al. [28] and subsequently studied by Dang et al. [29–32] which seemed to address all the inadequacies described above. However, the compatibility of the method with thioglycosides had to be verified. Upon application, and much to our delight, it was found that thioglycosides responded equally well compared to their *O*-glycosidic counterparts. It may be mentioned here that the response of thioglycosides towards this method was not reported in the original observation [28]. To the best of our knowledge, this method has not yet been applied directly on thioglycosides. So, having established an easy means to access D-*rhamno*-thioglycosides from their D-*manno-*counterparts, we set about devising efficient routes to reach the target trisaccharide. The following section bears elaboration of our efforts.

## Results and Discussion

**Scheme 1:**
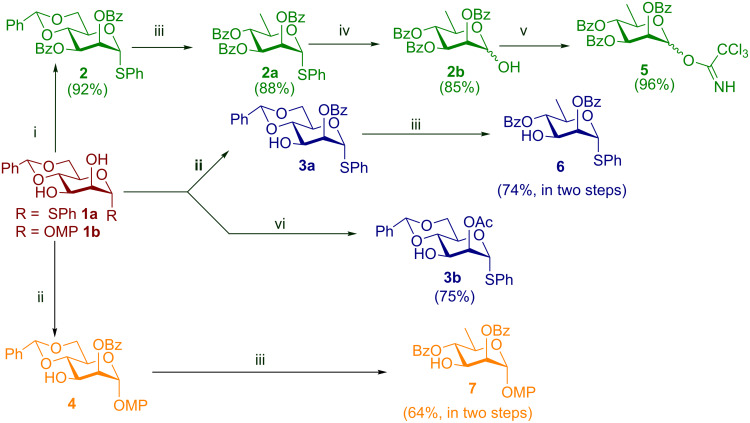
Preparation of the monomeric building blocks; reagents and conditions: i) Pyr., BzCl, 0 °C–rt; ii) PhC(OMe)_3_, CSA, MeCN, 0 °C–rt, 80% AcOH (aq), 25 °C; iii) DTBP, TIPST, octane, reflux; iv) TCCA, acetone–H_2_O, rt; v) CCl_3_CN, DBU, DCM; vi) MeC(OEt)_3_, CSA, MeCN, 0 °C–rt, 80% AcOH (aq), 25 °C.

Deoxygenation at the C-6 position of D-mannose derivatives bearing conventional or Crich’s modified 4,6-*O*-benzylidene protection [19–23] has been the principal philosophy behind the synthesis of the D-rhamnose [19–23,30,33–39] motif. This is a natural choice not only because it allows simultaneous selective blockage of the O-4 and O-6 positions but also sets up the model on which various deoxygenation protocols may be tried. The linkage pattern and stereochemistry at the glycosidic positions on the target dictate the presence of acyl protection on the O-2 position as shown in Scheme 1.

**Figure 2:**
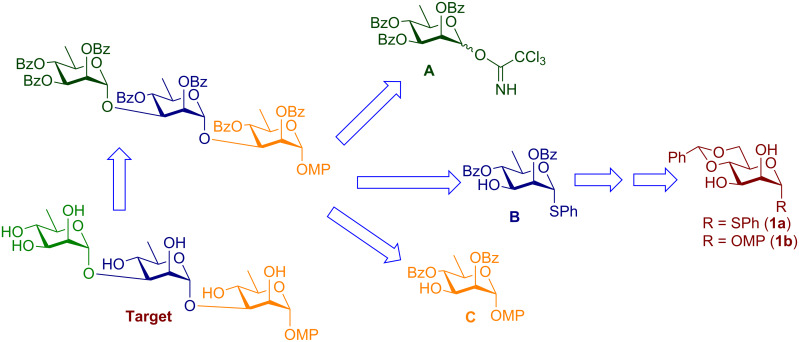
Retrosynthetic analysis.

We began our synthesis targeting two pivotal intermediates **1a** and **1b** which were obtained using previously reported methods [40–41] and then proceeding forward to the monomeric building blocks required according to the retrosynthetic analysis (Figure 2).

The procedures used and the results obtained to reach the intermediate targets have been summarized in Scheme 1. Accordingly, compounds **2** and **3b** [42] were obtained uneventfully from **1a** in 92% and 75%, respectively (Scheme 1). All attempts to place an acetyl protection at the O-2 position of **1b** with high yield failed. The poor yield was presumed to be due to the migration of the 2-*O*-acetyl group to the O-3 position leading to a mixture which was hard to separate. Hence we switched to *O*-benzoyl protection which was found to be less susceptible to migration [25]. The 2-*O-*benzoylated compound **4** was prepared by conversion of **1b** into its corresponding methyl 2,3-orthobenzoate derivative followed by the selective cleavage of the same with 80% aq AcOH. Compound **3a** which is the phenyl thioglycoside analogue of **4** was also accessed similarly. Both the compounds were next converted to their rhamnoside counterparts **6** and **7** [44], respectively by treatment with di-*tert*-butyl peroxide (DTBP) and triisopropylsilanethiol (TIPST) under reflux in octane over 2–3 h [30] in overall 74% and 64% yields, respectively, over two steps. It is worth noting here that during column chromatographic purification after AcOH-mediated cleavage of the orthobenzoate derivative some losses were incurred due to the migration of the 2-*O*-benzoyl group to the O-3 position. However the susceptibility of the benzoyl group to migration was much lower than that of its acetate analogue.

**Scheme 2:**
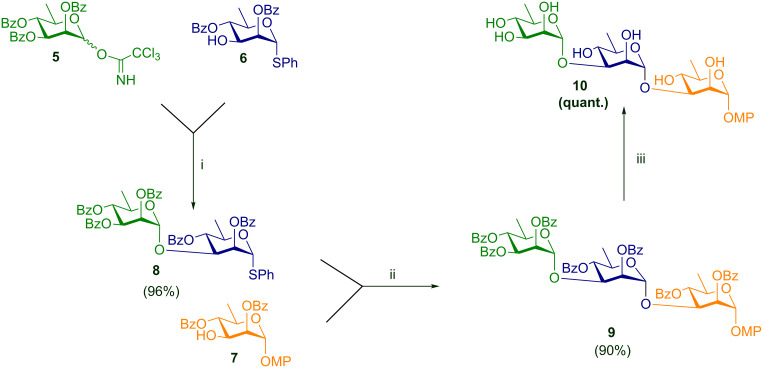
Sequential stepwise synthesis of the trisaccharide; reagents and conditions: i) TMSOTf, DCM, molecular sieves 4 Å, N_2_, −50 °C; ii) NIS, TMSOTf, DCM, molecular sieves 4 Å, N_2_, −10 °C; iii) NaOMe, MeOH, rt, 12 h.

The same problem persisted even in the rhamnosides **6** and **7**. Hence, to minimize the loss during purification, the chromatographic purification was reserved until the end of the deoxygenation step. Thus, the intermediate compounds **3a** and **4** were used without purification by column chromatography. Compound **2** was deoxygenated to its rhamnosyl counterpart **2a** in a high yield (88%). Next, the thioglycoside was hydrolyzed to the hemiacetal **2b** with trichloroisocyanuric acid (TCCA) in acetone–H_2_O [43] in 85% yield and was further converted almost quantitatively to the corresponding trichloroacetimidate **5** [44]. Having arrived at the monomeric building blocks **5**, **6**, and **7** we carried forward to the rhamnose-based disaccharide **8** which being a thioglycoside would subsequently serve as the glycosyl donor in the next step. Accordingly, **5** and **6** were coupled almost quantitatively to give **8**. The disaccharide so obtained was then coupled with **7** to give the protected trisaccharide **9** in 90% yield using a *N*-iodosuccinimide-trimethylsilyl trifluoromethanesulfonate (NIS-TMSOTf) combination as the activating reagent. The target trisaccharide was then obtained via deprotection under the Zémplen conditions to give **10** quantitatively, as is summarized in Scheme 2.

**Scheme 3:**
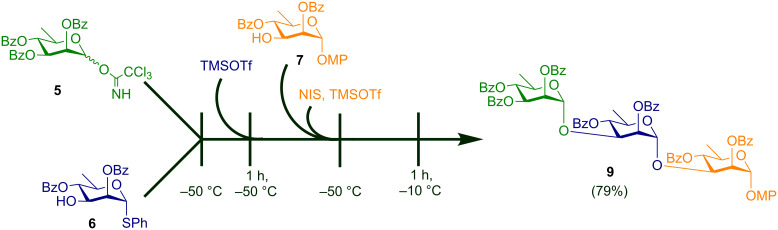
Synthesis of the trisaccharide by sequential one-pot glycosylation reactions; reagents and conditions: i) (a) TMSOTf, DCM, molecular sieves 4 Å, N_2_, −50 °C; (b) NIS, TMSOTf, −50 °C, DCM, molecular sieves 4 Å, N_2_, −10 °C.

However, in the interest of time and economy of resources consumed, a one-pot synthesis is always desirable. Accordingly, further optimization of the glycosylation protocol was achieved by carrying out the whole glycosylation process in one-pot leading to the target trisaccharide **9** in 79% yield (Scheme 3). In this case the disaccharide synthesis was set up as described previously, and then the second acceptor **7** was introduced into the reaction mixture at −50 °C after which NIS and TMSOTf were added successively at the same temperature. The temperature was then raised upto −10 °C, and the reaction mixture was stirred for another 1 h at that temperature to give the target trisaccharide as summarized in Scheme 3.

**Scheme 4:**
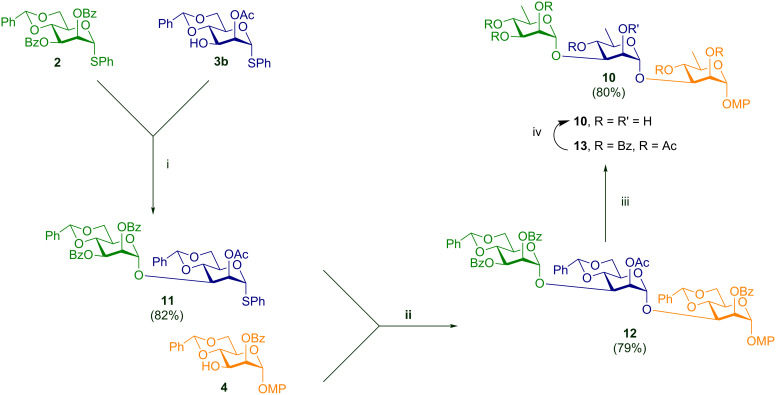
Synthesis of the target trisaccharide via global deoxygenation strategy; reagents and conditions**:** i) BSP, Tf_2_O, –60 °C, DCM, molecular sieves 4 Å, N_2_, –78 °C; ii) NIS, TMSOTf, DCM, molecular sieves 4 Å, N_2_, −10 °C; iii) DTBP, TIPST, octane, reflux, 3 h; iv) NaOMe, MeOH, rt, 12 h.

At this point, our search for overall synthetic efficiency was greatly augmented by a change of approach. Since, multiple deoxygenations had been observed by Dang et al. [30] on trehalose derivatives previously, we figured that we could access the rhamnose-based trisaccharide directly from a suitably derivatized *manno-*trisaccharide bearing the three 4,6-*O-*benzylidene protecting groups by a single global deoxygenation step. Accordingly, we coupled the monomeric units **2**, **3b** [45] and **4** to reach the mannose-based trisaccharide **12** (Scheme 4). The deoxygenation protocol, being incompatible with the *O-*benzyl protecting group, required an acyl protection profile on O-2 and O-3 positions. Such an arrangement was also in agreement with the stereochemical requirements at the anomeric positions of our target trisaccharide.

Since compounds **2** and **3b** bear structural similarity with each other it was expected that they would have similar reactivity towards glycosylative activation. Hence, a preactivation-based glycosylation protocol was devised for the first step leading to the disaccharide **11** using 1-benzenesulfinyl piperidine–triflic anhydride (BSP–Tf_2_O) [46]. The subsequent step was carried out using NIS–TMSOTf to give the mannose-based trisaccharide **12**. This trisaccharide was next deoxygenated globally to give the rhamnose-based trisaccharide **13** with high yield (80%). This was then deprotected under the Zémplen conditions to yield **10** quantitatively as is summarized in Scheme 4.

^1^H NMR in MeOH-*d*_4_ of the target trisaccharide **10** showed the anomeric protons of the consecutive rhamnose residues from the non-reducing end appearing at δ 5.09, 5.06 and 5.30 ppm, respectively. ^13^C NMR in the same solvent revealed that the chemical shifts of the anomeric carbons of the two consecutive rhamnose units from the non-reducing end coincided at δ 102.3 ppm (as also evidenced from HSQC data) with the corresponding ^1^*J*_C1-H1_ of 170.4 Hz and that of the anomeric carbon of the reducing end monomer unit appeared at 98.9 ppm with the corresponding ^1^*J*_C1-H1_ of 170.2 Hz. The value at δ 102.3 ppm can be corroborated with the reported [16] C_1_-chemical shifts at δ 103.19 and 103.43 with *^1^J*_C1-H1_ of 173 Hz, exhibited in D_2_O corresponding to the two [→3)-α-D-Rha*p*-(1→] units in the A-band polysaccharide of *P. aeruginosa*. The small difference in the chemical shifts and the corresponding ^1^*J*_C1-H1_ values may be attributed to the difference in the solvents (MeOH-*d*_4_ and D_2_O), chosen in these two cases for recording NMR. Comparing and considering the NMR data in these two cases we surmised that all the three anomeric carbons are α-configured [47].

## Conclusion

In short, we have described three different efficient routes to access the A-band trisaccharide associated with *P. aeruginosa*. The radical-based 6-deoxygenation protocol on 4,6-*O*-benzylidene (hitherto unexplored on thioglycosides) was utilized to arrive at the D-*rhamno*-thioglycoside derivatives from their D-*manno-*counterparts. This, along with a similar global 6-deoxygenation strategy on D-*manno-*trisaccharide derivative (bearing 4,6-*O-*benzylidene protection on each mannose unit) offer great potential in future oligosaccharide syntheses based on 6-deoxy hexoses.

## Supporting Information

File 1 Experimental details for the preparation of compounds **2a**, **2b**, **3a**, **4**, **6**–**13** and the corresponding characterization data.

File 2 ^1^H and ^13^C NMR of compounds **2a**, **4**, **6**, **8**–**13** and 2D NMR (COSY, HSQC and HMBC) of compound **10**.
