# Supplementary material for: Efficient routes toward the synthesis of the D-rhamno-trisaccharide related to the A-band polysaccharide of Pseudomonas aeruginosa
Source: Beilstein J Org Chem. 2014 Jul 1;10:1488–94. doi: 10.3762/bjoc.10.153 (PMC4142843; doi:10.3762/bjoc.10.153)
Supplement: File 1 — Experimental details for the preparation of compounds 2a, 2b, 3a, 4, 6–13 and the corresponding characterization data. [file Beilstein_J_Org_Chem-10-1488-s001.pdf]

Supporting Information File 1

for

Efficient routes toward the synthesis of the D-*rhamno*-trisaccharide related to the A-band polysaccharide of *Pseudomonas aeruginosa.*

Aritra Chaudhury, Sajal K. Maity and Rina Ghosh*

Address: Department of Chemistry, Jadavpur University, Kolkata 700 032, India

Email: Rina Ghosh* - ghoshrina@yahoo.com

*Corresponding author

Experimental details for the preparation of compounds 2a, 2b, 3a, 4, 6–13 and the corresponding characterization data

Table of contents

General remarks S2

Preparation of compounds and characterization data S2–S12

Procedure for the preparation of compound **9** via a one-pot glycosylation protocol S12

References S12

General remarks

For reactions under anhydrous conditions, all glasswares were stored in an oven and were flame-dried prior to use. All reagents and solvents were commercially available. Reagents were used without further purification, and solvents were distilled prior to use. Dry dichloromethane was obtained by distillation over phosphorous pentoxide. Thin layer chromatography (TLC) was done on glass plates coated with silica gel or on Merck silica gel plates (60-F_{254}) to monitor the reactions. Visualization of spots was accomplished by spraying the chromatograms with 5% ethanolic solution of sulfuric acid followed by charring on a hot-plate thereafter. 4 Å molecular sieves (MS) were activated by heating over a flame and then cooled under vacuum, prior to use. Column chromatography and flash column chromatography were performed using 60–120 and 230–400 mesh silica, respectively. Petroleum ether (PE, 60–80 °C) was used for chromatographic purpose. Melting points were recorded using a Toshniwal (India) melting point apparatus and are uncorrected. NMR spectra were recorded on a Bruker AV NMR spectrometer operating at 300 MHz and 500 MHz for ^{1}H NMR and at 75 MHz and 125 MHz for ^{13}C NMR in $CDCl_3$. Assignments were obtained using ^{1}H,^{1}H COSY, and ^{1}H,^{13}C HSQC and HMBC experiments. HRMS data were recorded on a Xevo G2 QTof mass spectrometer by electron spray ionization method. Specific rotations were measured on a Jasco P-1020 spectrometer. Rotations were determined using a cell of 1 dm path length.

Preparation of compounds and characterization data

4-Methoxyphenyl 2-*O*-benzoyl-4,6-*O*-benzylidene-α-D-mannopyranoside (4):

4-Methoxyphenyl 4,6-*O*-benzylidene-α-D-mannopyranoside (**1b**, 278 mg, 0.743 mmol) was stirred with trimethyl orthobenzoate (0.2 mL, 1.18 mmol) in acetonitrile (10 mL) at room

temperature for some time. Then 10-camphor sulfonic acid (CSA) (52 mg, 0.22 mmol) was added, and the reaction mixture was stirred at room temperature for 20 min. The solvent was then removed under reduced pressure, and the temperature was brought down to 0 °C, 80% aq acetic acid (4.6 mL) was then added, and the reaction was stirred at 0 ˚C for 10 min. The reaction mixture was quenched carefully with aq satd sodium bicarbonate solution. The product was extracted with dichloromethane (50 mL × 3), and the combined organic layer was washed with distilled water (100 mL × 1). The organic layer was dried over anhydrous sodium sulfate, and the solvent was removed under reduced pressure. The residue obtained was chromatographed (20% ethyl acetate/pet. ether) to give 4-methoxyphenyl 2-*O*-benzoyl-4,6-*O*-benzylidene-1-thio-α-D-mannopyranoside (242 mg, 68%) as a syrup.

^{1}H-NMR ($CDCl_3$, 300 MHz) (δ) 8.14-8.11 (d, 2H, *J* =7.6 Hz), 7.65-7.37 (m, 8H), 7.03-7.00 (d, 2H, *J* = 8.9 Hz), 6.86-6.83 (d, 2H, *J* =8.9 Hz), 5.67-5.66 (m, 2H), 5.56 (s, 1H) 4.58-4.54 (m, 1H), 4.30-4.26 (m, 1H), 4.16-4.09 (m, 2H), 3.91-3.85 (m, 1H), 3.78 (s, 3H).

^{13}C-NMR ($CDCl_3$, 75MHz) (δ) 166.0 (-*C*OPh), 155.4, 149.8, 137.08, 133.5, 129.9, 129.9, 129.5, 139.3, 128.5, 128.4, 126.3, 117.9, 114.7 (*C*-Ar), 102.3 (Ph*C*H-), 97.6 (*C-1*), 79.4, 72.5, 68.7, 67.4, 64.1 (*C2*-*C6*), 55.7 (*C*-OMe).

$[\alpha]_D^{25}$ 65.45 (c=1.26, $CHCl_3$).

HRMS (m/z): $[M + Li]^+$ calcd for $C_{27}H_{26}O_8Li$ 485.1788 found 485.1783.

Compound **3a** was also prepared similarly and characterization data was found to be in agreement with the literature data [1].

General procedure for the preparation of compounds 2a, 6 and 7:

Phenyl 2,3,4-tri-*O*-benzoyl-1-thio-α-D-rhamnopyranoside (2a):

Phenyl 2,3-di-*O*-benzoyl-4,6-*O*-benzylidene-1-thio-α-D-mannopyranoside (**2**, 1.27 g, 2.25 mmol) was taken along with octane (20 mL), and di-*tert*-butylperoxide (0.42 mL, 2.25 mmol) and tri-isopropylsilanethiol (0.05 mL, 0.25 mmol) were added. The reaction mixture was refluxed under argon atmosphere for 2 h. TLC at this point showed the reaction to be complete. The octane was removed under reduced pressure, and the residue was purified by flash chromatography (15% ethyl acetate/pet. ether) to yield the product **2a** (1.12 g, 88%) as a white foamy solid.

Characterization data:

Phenyl 2,3,4-tri-*O*-benzoyl-1-thio-α-D-rhamnopyranoside (2a):

^{1}H-NMR ($CDCl_3$, 300 MHz) (δ) 8.11-8.08 (d, 2H, *J* = 7.2 Hz), 8.03-8.01 (d, 2H, *J* = 7.3 Hz), 7.87 -7.84 (d, 2H, *J* = 7.3 Hz), 7.63-7.25 (m, 14H), 5.95 (bs, 1H), 5.86-5.77 (m, 2H), 4.75-4.66 (m, 1H), 1.42-1.40 (d, 3H, *J* = 6.2 Hz).

^{13}C-NMR ($CDCl_3$, 75 MHz) (δ) 165.8, 165.5 (-*C*OPh), 133.5-127.9 (*C*-Ar), 85.9 (*C-1*), 72.4, 71.9, 70.4, 68.2 (*C2*-*C5*), 17.6 (*C*-*6*).

$[\alpha]_D^{25}$ -34.69 (c=1.26, $CHCl_3$).

HRMS (m/z): $[M + Na]^+$ calcd for $C_{33}H_{28}O_7SNa$ 591.6260 found 591.6253.

Phenyl 2,4-di-*O*-benzoyl-1-thio-α-D-rhamnopyranoside (6):

^{1}H-NMR ($CDCl_3$, 300 MHz) (δ) 8.12-8.09 (m, 4H), 7.64-7.29 (m, 11H), 5.67-5.66 (m, 2H), 5.37-5.31 (t, 1H, *J* = 9.7 Hz), 4.61-4.54 (m, 1H),4.33-4.29 (dd, 1H, *J* = 3 Hz and 9.8 Hz) 1.37-1.35 (d, 3H, *J* = 6.2 Hz).

^{13}C-NMR ($CDCl_3$, 75 MHz) (δ) 167.2, 165.9 (-*C*OPh), 133.6-127.8 (*C*-Ar), 85.9 (*C-1*), 75.7, 74.9, 69.7, 67.6 (*C2*-*C5*), 17.6 (*C*-*6*).

$[\alpha]_D^{25}$ 106 (c=1.9, $CHCl_3$).

HRMS (m/z): $[M + Na]^+$ calcd for $C_{26}H_{24}O_6SNa$ 487.1191 found 487.1171.

4-Methoxyphenyl 2,4-di-*O*-benzoyl-1-thio-α-D-rhamnopyranoside (7):

^{1}H-NMR ($CDCl_3$, 300 MHz) (δ) 8.15-8.13 (d, 2H, *J* = 7 Hz), 8.10-8.07 (d, 2H, *J* = 9Hz),7.64-7.44 (m, 6H) 7.07-7.04 (d, 2H, *J* = 8.9 Hz), 6.87-6.84 (d, 2H, *J* = 8.9 Hz), 5.58-5.57 (m, 2H), 5.38-5.29 (m, 1H) 4.54-4.50 (dd, 1H, *J* = 2.4 Hz and 9.8 Hz), 4.28-4.22 (m, 1H), 3.79 (s, 3H, -OMe), 1.32-1.31 (d, 6H, *J* = 6.2 Hz).

^{13}C-NMR ($CDCl_3$, 75 MHz) (δ) 167.1, 166.0 (-*C*OPh), 155.3, 150.2, 133.6-128.5 (*C*-Ar), 117.7, 114.7, 96.5(*C-1*), 75.5, 73.1, 68.9, 66.9 (*C2*-*C5*), 55.7 (*C*-OMe), 17.7 (*C*-*6*).

The data was found to be in agreement with that of the reported literature [2].

2,3,4-tri-*O*-benzoyl-D-rhamnopyranose (2b):

Compound **2a** (345 mg, 0.607 mmol) was dissolved in acetone-H_2O (4:1, 10 mL), and TCCA (142 mg, 0.61 mmol) was added. The reaction mixture was stirred at room temperature for 45 min. TLC (20% ethyl acetate/pet. ether) at this point showed the reaction to be complete. The solvent was concentrated under reduced pressure, and the crude residue was dissolved in dichloromethane. The organic layer was washed successively with aq $NaHCO_3$ (200 mL × 1) and brine (200 mL × 1). The organic layer was dried over anhydrous sodium sulfate, and the solvent was removed under reduced pressure. The residue obtained was chromatographed (20% ethyl acetate/pet.ether) to give the product **2b** (245 mg, 85%) as a white solid (m.p. 203–205 °C).

^{1}H-NMR ($CDCl_3$, 300 MHz) (δ) 8.13-7.24 (m, 15H), 5.96-5.92 (m, 1H), 5.73-5.67 (m, 2H), 5.47 (s, 1H), 4.52-4.43 (m, 1H), 3.81-3.77(bs, 1H), 1.37 (d, 3H, *J* = 6.19 Hz).

^{13}C-NMR ($CDCl_3$, 75 MHz) (δ) 165.8, 165.5, 165.4 (-*C*OPh), 133.5-128.2 (*C*-Ar), 92.4 (*C-1*), 71.9, 71.2, 69.6, 66.8 (*C2*-*C5*), 17.6 (*C*-*6*).

The data was found to be in agreement with that of the reported literature [3].

Phenyl 2,3,4-tri-*O*-benzoyl-α-D-rhamnopyranosyl-(1→3)-2′,4′-di-*O*-benzoyl-1-thio-α-D-rhamnopyranoside (8):

A solution of compounds **5** (72 mg, 0.12 mmol**)** and **6** (54 mg, 0.12 mmol) in dichloromethane (4 mL) was stirred with freshly activated molecular sieves 4 Å at room temperature under argon atmosphere for 15 min. Then the temperature was lowered to -50 °C, and TMSOTf (9 µl, 0.04 mmol) was added. The reaction mixture was stirred for 2 h at which point TLC showed the reaction to be complete. The reaction mixture was quenched with triethylamine, diluted with dichloromethane, and the molecular sieves were filtered over a celite bed under suction. The filtrate was washed successively with aq satd sodium bicarbonate (100 mL × 1) and brine (100 mL × 1). The organic layer was dried over anhydrous sodium sulfate. The solvent was removed under reduced pressure, and the crude material so obtained was purified by flash chromatography (20% ethyl actetate/ pet. ether) to give the product **8** (102 mg, 96%) as a syrup.

^{1}H-NMR ($CDCl_3$, 500 MHz) (δ) 8.24-8.22 (d, 2H, *J* = 7.5 Hz), 8.16-8.15 (d, 2H, *J* = 7.5 Hz), 7.93-7.91 (d, 2H, *J* = 7.5 Hz), 7.75-7.18 (m, 24H), 5.79-5.78 (d, 1H, *J* = 1.5 Hz), 5.73-5.72 (d, 1H, *J* = 1.0 Hz), 5.69-5.66 (t, 1H, *J* = 9.5 Hz), 5.62-5.5.59 (dd, 1H, *J* = 3.5 Hz and 10.0 Hz), 5.53-5.49 (t, 1H, *J* = 10.0 Hz), 5.29 (s, 1H), 5.26 (s, 1H), 4.59-4.53 (m, 1H), 4.49-4.47 (dd, 1H, *J* = 3.0 Hz and 9.5 Hz), 4.18-4.12 (m, 1H), 1.40-1.39 (d, 3H, *J* = 6.5 Hz), 1.19-1.18 (d, 3H, *J* = 6.5 Hz).

^{13}C-NMR ($CDCl_3$, 125 MHz) (δ) 166.2, 165.9, 165.7, 165.1 (-*C*OPh), 133.7-128.0 (*C-Ar*), 99.5, 85.8, 76.7, 74.0, 73.3, 71.7, 70.8, 69.5, 68.5, 67.8, 17.8 (*C*-Me), 17.5 (*C*-Me).

$[\alpha]_D^{25}$ 50.85 (c=1.06, $CHCl_3$).

HRMS (m/z): $[M + Na]^+$ calcd for $C_{53}H_{46}O_{13}SNa$ 945.2557 found 945.2552.

4-Methoxyphenyl 2,3,4-tri-*O*-benzoyl-α-D-rhamnopyranosyl-(1→3)-2′,4′-di-*O*-benzoyl-α-D-rhamnopyranosyl-(1→3)-2″,4″-di-*O*-benzoyl-α-D-rhamnopyranoside (9):

A solution of compounds **8** (75 mg, 0.08 mmol**)** and **7** (40 mg, 0.08 mmol) in dichloromethane (4 mL) was stirred with freshly activated molecular sieves 4 Å at room temperature under inert atmosphere for 15 min. Then the temperature was lowered to -10 °C, and *N*-iodosuccinimide (21 mg, 0.1 mmol) was added followed by TMSOTf (3 μl, 0.02 mmol). The reaction mixture was stirred for 1 h at which point TLC showed the reaction to be complete. The reaction mixture was diluted with dichloromethane, and the molecular sieves were filtered over a celite bed under suction. The filtrate was washed successively with aq satd $NaHCO_3$ (100 mL × 1) and brine (100 mL × 1). The organic layer was dried over anhydrous sodium sulfate. The solvent was removed under reduced pressure, and the crude material so obtained was purified by flash chromatography (25% ethyl actetate/ pet. ether) to give the product **9** (94 mg, 90%) as a syrup.

^{1}H-NMR ($CDCl_3$, 500 MHz) (δ) 8.24-8.21 (d, 2H, *J* = 6.8 Hz), 8.10-8.08 (m, 4H), 7.92-7.86 (m, 4H) (-*C*OPh), 7.70-7.15 (m, 25H) (*C*-Ar), 7.08-7.05 (d, 2H, *J* = 9.0 Hz), 6.87-6.84 (d, 2H, *J* = 9.0 Hz), 5.71 (m, 1H), 5.67-5.62 (m, 2H), 5.50-5.5.40 (m, 2H), 5.36-5.30 (m, 2H), 5.19-5.18 (m, 2H), 4.86 (bs, 1H), 4.68-4.64 (m, 1H), 4.28-4.19 (m, 2H), 4.04-3.95 (m, 1H), 3.86-3.78 (m, 4H), 1.33-1.31 (d, 3H, *J* = 6.2 Hz), 1.14-1.12 (d, 3H, *J* = 6.3 Hz), 0.76-0.74 (d, 3H, *J* = 6.2 Hz).

^{13}C-NMR (CDCl$_3$, 125 MHz) (δ) 165.9, 165.7, 165.6 (-*C*OPh), 155.3, 150.1, 133.3-128.0 (*C*-Ar), 117.7, 114.7, 99.2, 96.4, 77.2, 75.6, 72.9, 72.4, 72.2, 71.6, 70.5, 69.4, 67.8, 67.4, 67.1, 55.7, 17.7 (*C*-Me), 17.5 (*C*-Me), 16.9 (*C*-Me).

$[\alpha]_D^{25}$ -109.48 (c=1.06, CHCl$_3$).

HRMS (m/z): $[M + Na]^+$ calcd for $C_{74}H_{66}O_{21}Na$ 1313.3994 found 1313.3991.

Phenyl 2,3-di-*O*-benzoyl-4,6-*O*-benzylidene-α-D-mannopyranosyl-(1→3)- 2′-*O*-acetyl-4′,6′-*O*-benzylidene-1-thio-α-D-mannopyranoside (11).

Compound **2** (170 mg, 0.3 mmol) was dissolved in dichloromethane (4 mL) and 1-benzenesulfinylpiperidine (69 mg, 0.33 mmol) was added. The resulting solution was stirred at room temperature with molecular sieves 4 Å under nitrogen atmosphere for 15 min. It was then stirred at −60 °C with triflic anhydride (0.06 mL, 0.36 mmol) for 20 min. The temperature was lowered to −78 °C, and compound **3b (**132 mg, 0.3 mmol**)** was added as a solution in dichloromethane. The reaction mixture was stirred at the same temperature for 45 min at which point the reaction was found to be complete. The reaction was quenched with triethylamine (0.1 mL, 0.7 mmol), and then the temperature was raised to room temperature. The reaction mixture was diluted with dichloromethane and filtered over celite to remove the molecular sieves. The bed was washed with dichloromethane, and the combined filtrate and washings was washed successively with aq satd sodium bicarbonate (100 mL × 1) and brine (100 mL × 1). The organic layer was then dried over anhydrous sodium sulfate. The solvent was removed under reduced pressure, and the residue was purified by flash chromatography (22.5% ethyl acetate/pet. ether) to yield the product **11** (211 mg, 82%).

^{1}H-NMR (CDCl$_3$, 500 MHz) (δ) 8.10-8.08 (d, 2H, *J* = 10 Hz), 7.95-7.94 (d, 2H, *J* = 10 Hz) 7.62-7.23 (m, 21H), 5.84-5.5.83 (m, 1H), 5.73-5.66 (m, 4H), 5.51 (s, 1H), 5.45 (s, 1H), 4.42-4.16 (m, 7H), 3.94-3.91 (m, 2H).

^{13}C-NMR (CDCl$_3$, 125 MHz) (δ) 170.0, 165.4 (-*C*OPh), 137.1-125.9 (*C*-Ar), 102.0 (Ph*C*H-), 101.3 (Ph*C*H-), 99.6, 87.1, 79.0, 76.6, 72.8, 72.1, 70.6, 68.8, 68.3, 64.9, 64.7, 20.9 (*C*-OAc).

$[\alpha]_D^{25}$ -6.27 (c=1.03, CHCl$_3$).

HRMS (m/z): $[M + Na]^+$ calcd for $C_{48}H_{44}O_{13}SNa$ 883.2400 found 883.2395.

4-Methoxyphenyl 2,3-di-*O*-benzoyl-4,6-*O*-benzylidene-α-D-mannopyranosyl-(1→3)-2′-*O*-acetyl-4′,6′-*O*-benzylidene-α-D-mannopyranosyl-(1→3)-2″-*O*-benzoyl-4″,6″-*O*-benzylidene-α-D-mannopyranoside (12):

A solution of compounds **4** (306 mg, 0.35 mmol) and **11** (153 mg, 0.35 mmol) in dichloromethane (10 mL) was stirred with freshly activated molecular sieves 4 Å at room temperature under inert atmosphere for 15 min. Then the temperature was lowered to −10 °C, and *N*-iodosuccinimide (84 mg, 0.35 mmol) was added followed by TMSOTf (6.5 μl, 0.04 mmol). The reaction mixture was stirred for 1 h at which point TLC showed the reaction to be complete. The reaction mixture was diluted with dichloromethane, and the molecular sieves were filtered over a celite bed under suction. The bed was washed with dichloromethane, and the combined filtrate and washings was washed successively with aq satd sodium bicarbonate (250 mL × 1) and brine (250 mL × 1). The organic layer was dried over anhydrous sodium sulfate. The solvent was removed under reduced pressure, and the crude material so obtained was purified by flash chromatography (20% ethyl actetate/pet. ether) to give the product **12** (344 mg, 79%) as a white solid.

^{1}H-NMR (CDCl$_3$, 300 MHz) (δ) 8.17-8.14 (d, 2H, *J* = 7.6 Hz), 8.04-8.01 (d, 2H, *J* = 7.6 Hz), 7.89-7.86 (d, 2H, *J* = 7.7 Hz), 7.65-7.22 (m, 24H), 7.04-7.01 (d, 2H, *J* = 8.9 Hz), 6.87-6.84 (d, 2H,

J = 8.9 Hz), 5.78-5.74 (m, 3H), 5.66-5.62 (m, 2H), 5.57 (s, 1H), 5.55 (s, 1H), 5.45 (s, 1H), 5.35-5.34 (m, 2H), 4.68-4.65 (m, 1H), 4.41-4.05 (m, 10H), 3.95-3.84 (m, 2H), 3.79 (s, 3H), 3.75-3.72 (m, 1H), 3.66-3.59 (t, 1H, *J* = 10.2 Hz), 2.27 (s, 3H).

^{13}C-NMR ($CDCl_3$, 75 MHz) (δ) 165.9, 165.5, 165.1, 165.0 (-*C*OPh), 155.5, 149.6, 137.2-126.0 (*C*-Ar), 118.1, 114.8, 101.9 (Ph*C*H-), 101.5 (Ph*C*H-), 101.3 (Ph*C*H-), 99.4, 99.1, 97.8, 79.3, 79.0, 77.2, 71.8, 70.9, 70.8, 70.6, 68.8, 68.6, 64.3, 64.2, 55.7, 20.8 (*C*-OAc).

$[\alpha]_D^{25}$ -35.4 (c=1.06, $CHCl_3$).

HRMS (m/z): $[M + Na]^+$ calcd for $C_{69}H_{64}O_{21}SNa$ 1251.3838 found 1251.3835.

4-Methoxyphenyl 2,3,4-tri-*O*-benzoyl-α-D-rhamnopyranosyl-(1→3)-2′-*O*-acetyl-4′-*O*-benzoyl-α-D-rhamnopyranosyl-(1→3)-2″4″-di-*O*-benzoyl–α–D-rhamnopyranoside (13):

The trisaccharide **12** (110 mg, 0.09 mmol) was taken along with octane (5 mL) and di-*tert*-butyl peroxide (0.06 mL, 0.27 mmol), and tri-isopropylsilanethiol (6.0 μl, 0.03 mmol) was added. The reaction mixture was refluxed under argon atmosphere for 3 h. TLC at this point showed the reaction to be complete. The octane was removed under reduced pressure, and the residue was purified by flash column chromatography (22% ethyl acetate/pet. ether) to yield the product **13** (95 mg, 80%) as a white solid.

^{1}H-NMR ($CDCl_3$, 300 MHz) (δ) 8.21-8.19 (d, 2H, *J* = 7.7 Hz), 8.07-8.04 (d, 2H, *J* = 7.8 Hz), 7.94-7.91 (d, 2H, *J* = 7.7 Hz), 7.86-7.83 (d, 2H, *J* = 7.9 Hz), 7.81-7.78 (d, 2H, *J* = 7.9 Hz), 7.71-7.68 (d, 2H, *J* = 7.9 Hz), 7.59-7.16 (m, 18H), 7.08-7.05 (d, 2H, *J* = 8.9 Hz), 6.87-6.85 (d, 2H, *J* = 7.9 Hz), 5.68-5.15 (m, 4H), 5.44-5.38 (m,1H), 5.34-5.27 (m,1H), 5.21 (s, 1H), 5.12 (s, 1H), 5.04 (s, 1H), 4.86 (s, 1H), 4.64-4.60 (m,1H), 4.21-4.09 (m, 2H), 3.93-3.92 (m, 2H), 3.78 (s, 3H), 2.14 (s, 3H), 1.32-1.30 (d, 3H, *J* = 5.8 Hz), 1.13-1.10 (d, 3H, *J* = 5.7 Hz), 0.95-0.93 (d, 3H, *J* = 5.8 Hz).

^{13}C-NMR ($CDCl_3$, 75 MHz) (δ) 169.9, 166.0, 165.8, 165.7, 165.4, 164.9 , 164.6 (-*C*OPh*)*, 155.3, 150.1, 133.6-128.1 (*C*-Ar), 99.4, 98.7, 77.2, 73.7, 73.0, 72.9, 72.2, 71.7, 71.3, 70.5, 69.1, 67.8, 67.2, 55.7, 20.8 (*C*-OAc), 17.7 (*C*-Me), 17.3 (*C*-Me), 17.1(*C*-Me).

$[\alpha]_D^{25}$ -105.5 (c= 0.96, $CHCl_3$).

HRMS (m/z): $[M + Na]^+$ calcd for $C_{69}H_{64}O_{21}SNa$ 1251.3838 found 1251.3828.

4-Methoxyphenyl-α-D-rhamnopyranosyl-(1→3)-α-D-rhamnopyranosyl-(1→3)-α–D-rhamnopyranoside (10):

The trisaccharide **13** (134 mg, 0.11 mmol) was taken in freshly dried methanol (14 mL) and a 1 M sodium methoxide–methanol solution (1.5 mL) was added at 0 °C. The reaction mixture was allowed to attain room temperature and then stirred for 12 h. After the completion of the reaction the mixture was stirred with Dowex 50W (H^+) resin (activated) for 15 min to neutralize the basic solution. The resin was filtered under suction and the resin was washed thoroughly with methanol. The combined filtrate and washings was collected in a round bottom flask, and the solvent was removed under reduced pressure to yield the product (61 mg, quantitative) as a white solid.

^{1}H-NMR (D_4-MeOH, 500 MHz) (δ) 7.01 (d, 2H, *J* = 9.5 Hz), 6.86 (d, 2H, *J* = 9.0 Hz), 5.30 (s, 1H, *H-1*), 5.09 (s, 1H, *H-1''*), 5.06 (s, 1H, *H-1'*), 4.13 (s, 1H, *H-2''*), 4.10 (s, 1H, *H-2*), 4.02 (s, 1H, *H-2'*), 3.96-3.81 (m, 5H), 3.74-3.72 (m, 4H), 3.62 (t, 1H, *J* = 9.5 Hz, *H-4'*), 3.56 (t, 1H, *J* = 9.5 Hz, *H-4''*), 3.42 (t, 1H, *J* = 9.5Hz, *H-4*), 1.31 (d, 3H, *J* = 5.5 Hz, *H-6'*), 1.28 (d, 3H, *J* = 7.5 Hz, *H-6*), 1.25 (d, 3H, *J* = 6.0 Hz, *H-6''*).

^{13}C-NMR (D_4-MeOH, 125 MHz) (δ) 154.7, 149.9, 117.3, 113.9 (*C*-Ar), 102.3 (*C-1', C-1''*), 98.9 (*C-1*), 78.2 (*C-3''*), 77.6 (*C-3*), 72.3 (*C-4'*), 71.4 (*C-4''*), 71.3 (*C-4*), 70.4 (*C-3', C-2'*), 70.2 (*C-2,C-2''*), 69.0 (*C-5*), 68.6 (*C-5''*), 68.3 (*C-5'*), 54.3 (*C*-OMe), 16.3 (*C-6, C-6'*), 16.2 (*C-6''*).

$[\alpha]_D^{25}$ 134.45 (c=1.4, MeOH).

HRMS (m/z): $[M + Na]^+$ calcd for $C_{25}H_{38}O_{14}Na$ 585.2159 found 585.2157.1

Procedure for the preparation of Compound 9 via a one-pot glycosylation protocol:

Compounds **5** (62 mg, 0.1 mmol) and **6** (47 mg, 0.1 mmol) were taken in dichloromethane (4 mL) along with freshly activated molecular sieves 4 Å, and the mixture was stirred at room temperature for 15 min. The temperature was next lowered to −50 °C, and TMSOTf (9 μl, 0.05 mmol) was added. The reaction mixture was stirred at the same temperature for 1 h. Compound **7** (43 mg, 0.09 mmol) was added followed by NIS (26 mg, 0.12 mmol) and TMSOTf (2 μl, 0.01 mmol) at −50 °C. The temperature was raised up to −10 °C and stirred for 1 h. TLC (25% ethyl actetate/pet. ether) showed the reaction to be complete at this point. The reaction mixture was quenched with Et_3N and then filtered over a celite bed under suction. The bed was washed with dichloromethane, and the combined filtrate and washings were washed successively with aq $NaHCO_3$ (100 mL × 1) and brine (100 mL × 1). The organic layer was dried over anhydrous sodium sulfate. The solvent was removed under reduced pressure, and the crude material so obtained was purified by flash chromatography (25% ethyl acetate/pet. ether) to give the product **9** (100 mg, 79%) as a syrup.

References

1. Franzyk, H.; Meldal, M.; Paulsen, H.; Bock, K. *J. Chem. Soc. Perkin Trans. 1* **1995**, *22,* 2883-2898.
2. Fauré, R.; Shiao, T. C.; Damerval, S.; Roy, R. *Tetrahedron Lett.* **2007,** *48,* 2385–2388.
3. Faur´e, R.; Shiao, T. C.; Lagnoux, D.; Gigu`ere, D.; Roy, R. *Org. Biomol. Chem.* **2007**, *5,* 2704–2708.
