# Supplementary material for: Efficient routes toward the synthesis of the D-rhamno-trisaccharide related to the A-band polysaccharide of Pseudomonas aeruginosa
Source: Beilstein J Org Chem. 2014 Jul 1;10:1488–94. doi: 10.3762/bjoc.10.153 (PMC4142843; doi:10.3762/bjoc.10.153)

Supporting Information File 2

for

Efficient routes toward the synthesis of the D-*rhamno*-trisaccharide related to the A-band polysaccharide of *Pseudomonas aeruginosa.*

Aritra Chaudhury, Sajal K. Maity and Rina Ghosh*

Address: Department of Chemistry, Jadavpur University, Kolkata 700 032, India

Email: Rina Ghosh* - ghoshrina@yahoo.com

*Corresponding author

^{1}H and ^{13}C NMR of compounds 2a, 4, 6, 8–13 and 2D NMR (COSY, HSQC and HMBC) of compound 10

Table of Contents

1H and ^{13}C NMR of compound **2a**	S2
1H and ^{13}C NMR of compound **4**	S3
1H and ^{13}C NMR of compound **6**	S4
1H and ^{13}C NMR of compound **8**	S5
1H and ^{13}C NMR of compound **9**	S6
1H and ^{13}C NMR of compound **11**	S7
1H and ^{13}C NMR of compound **12**	S8
1H and ^{13}C NMR of compound **13**	S9
1H and ^{13}C NMR of compound **10**	S10
1H-1H COSY spectra of compound **10**	S11–S12
HSQC spectra of compound **10**	S13–S15
HMBC spectra of compound **10**	S16–21

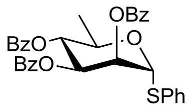

2a
(^{1}H NMR)

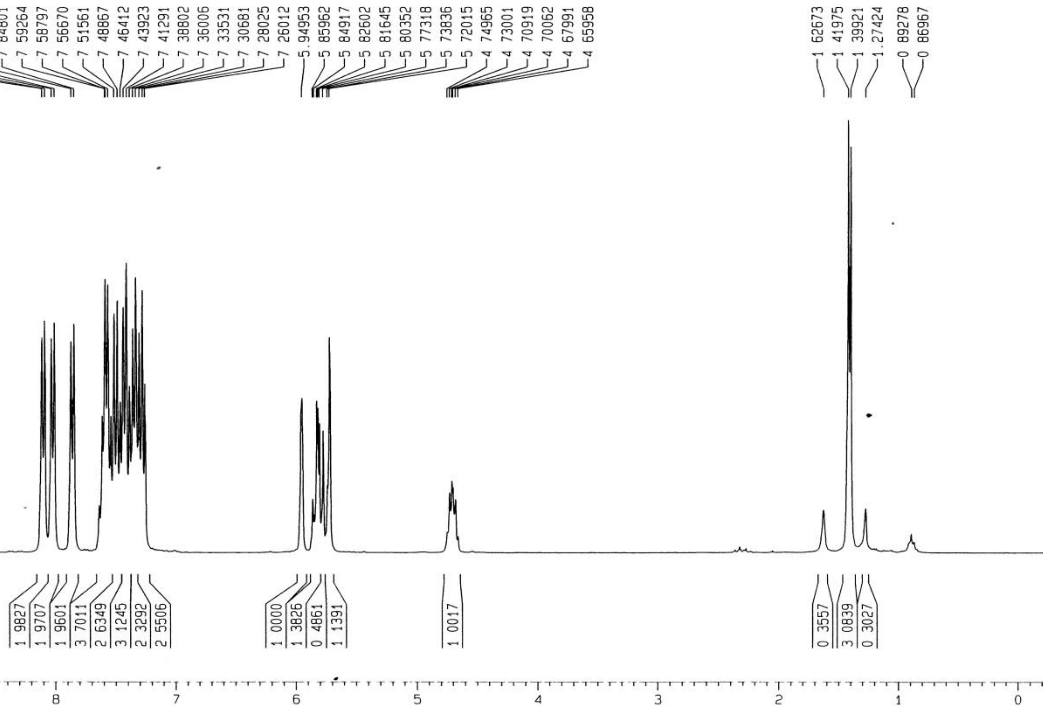

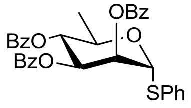

2a
(^{13}C NMR)

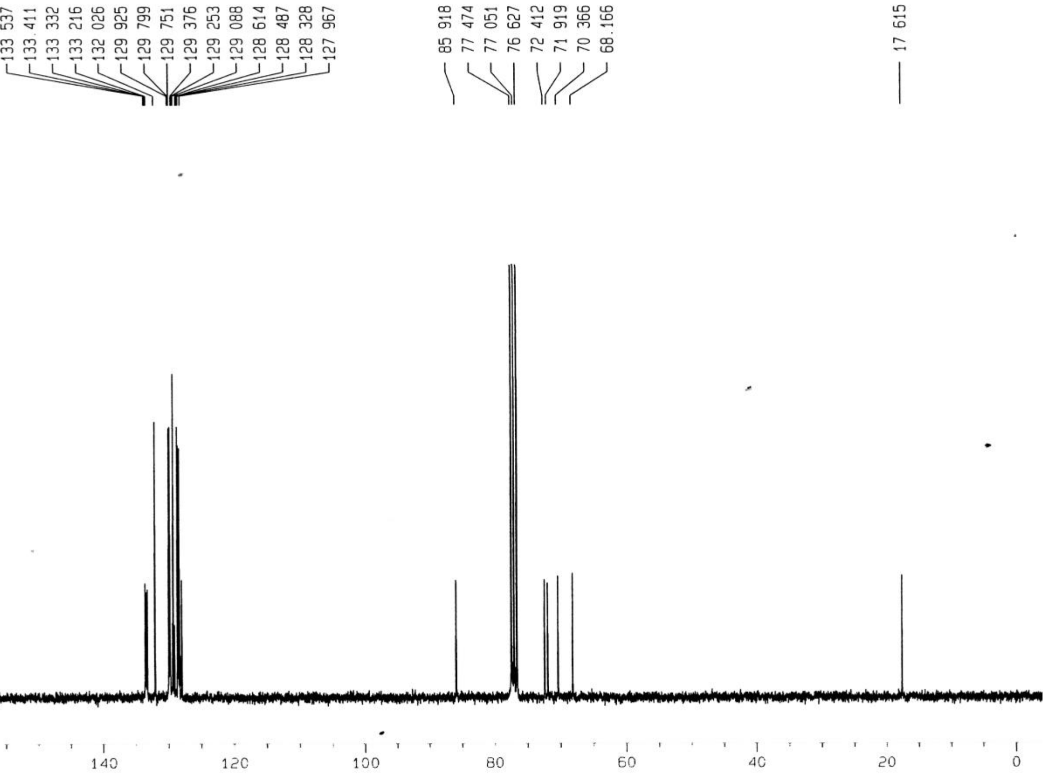

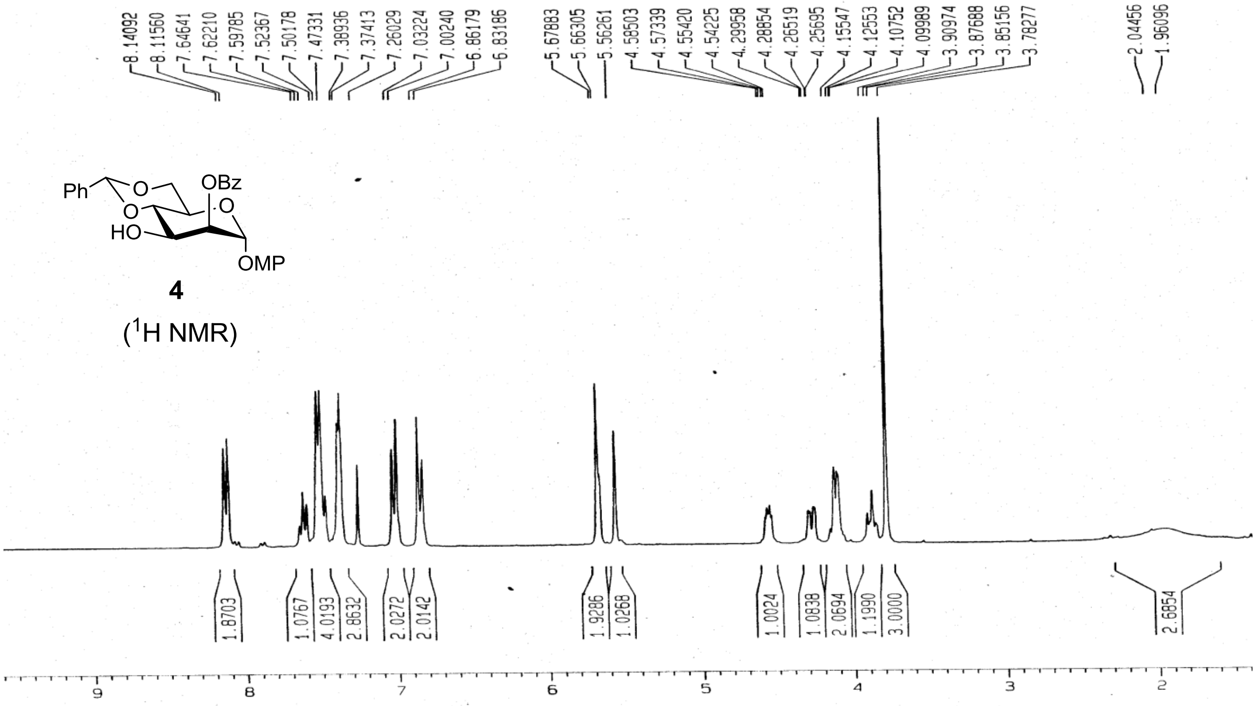
8.14092
8.11560
7.64641
7.62210
7.59785
7.52367
7.50178
7.47331
7.38936
7.37413
7.26029
7.03224
7.00240
6.86179
6.83186
5.67883
5.66305
5.56261
4.58503
4.57339
4.55420
4.54225
4.29958
4.28854
4.26519
4.25695
4.15547
4.12553
4.10752
4.09989
3.90974
3.87688
3.85156
3.78277
2.04456
1.96096
Ph
O
O
OBz
O
HO
OMP
4
(¹H NMR)
1.8703
1.0767
4.0193
2.8632
2.0272
2.0142
1.9286
1.0268
1.0024
1.0838
2.0694
1.1990
3.0000
2.6854
9
8
7
6
5
4
3
2

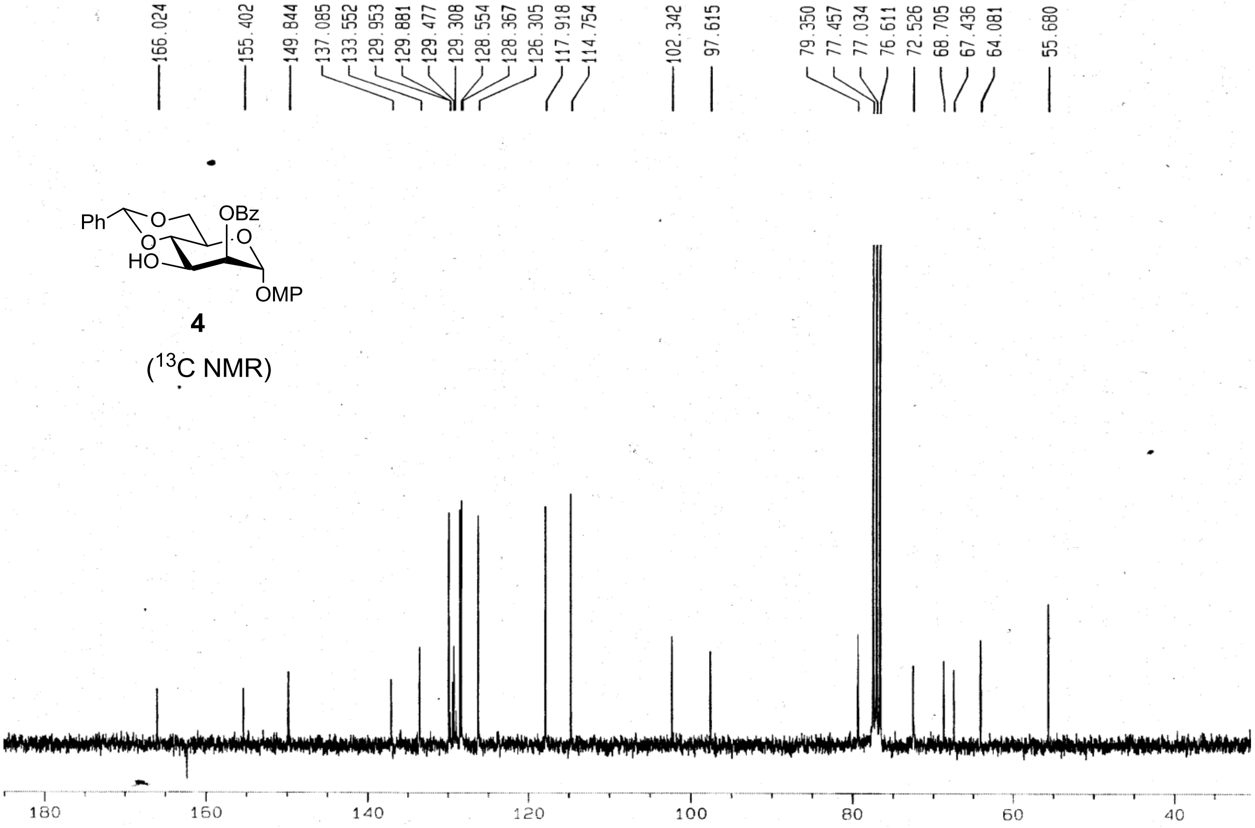
166.024
155.402
149.844
137.085
133.552
129.953
129.881
129.477
129.308
128.554
128.367
126.305
117.918
114.754
102.342
97.615
79.350
77.457
77.034
76.611
72.526
68.705
67.436
64.081
55.680
Ph
O
O
OBz
O
HO
OMP
4
(¹³C NMR)
180
160
140
120
100
80
60
40

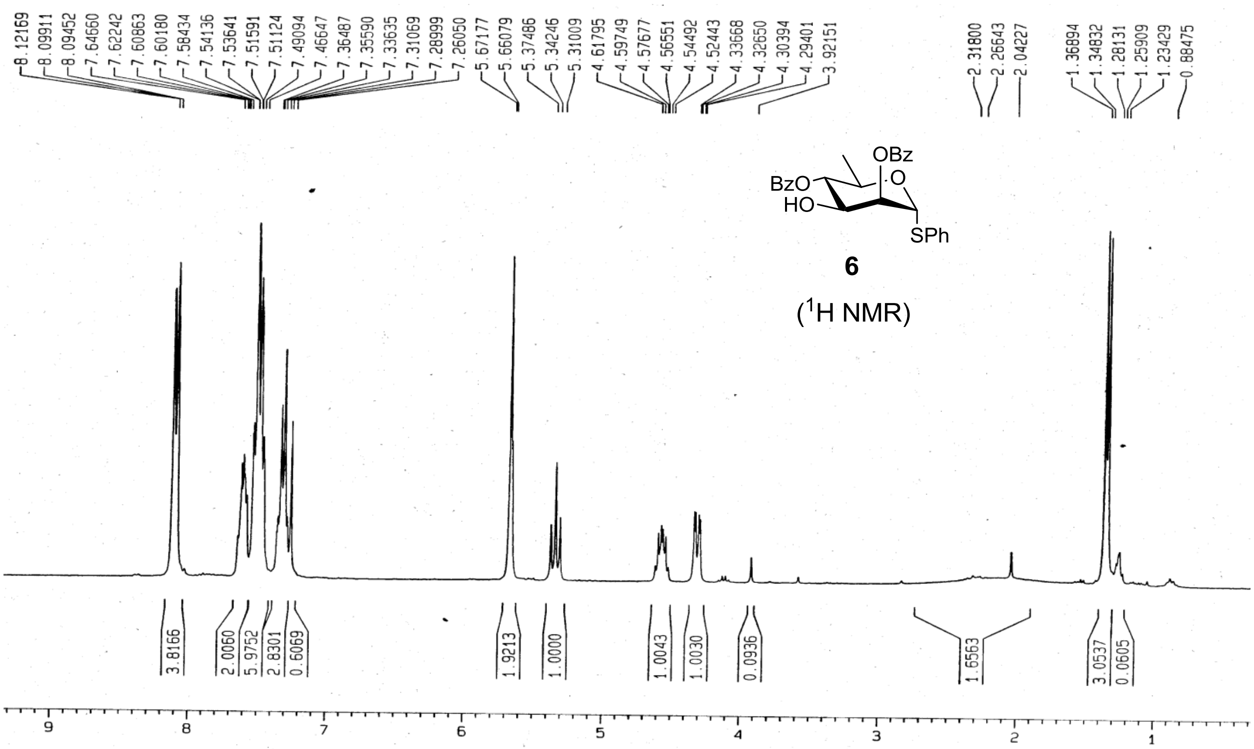
8.12169
8.09911
8.09452
7.64660
7.62242
7.60863
7.60180
7.58434
7.54136
7.53641
7.51591
7.51124
7.49094
7.46647
7.36487
7.35590
7.33635
7.31069
7.28999
7.26050
5.67177
5.66079
5.37486
5.34246
5.31009
4.61795
4.59749
4.57677
4.56551
4.54492
4.52443
4.33668
4.32650
4.30394
4.29401
3.92151
2.31800
2.26643
2.04227
1.36894
1.34832
1.28131
1.25909
1.23429
0.88475
OBz
BzO
HO
O
SPh
6
(^{1}H NMR)
3.8166
2.0060
5.9752
2.8301
0.6069
1.9213
1.0000
1.0043
1.0030
0.0936
1.6563
3.0537
0.0605
9
8
7
6
5
4
3
2
1

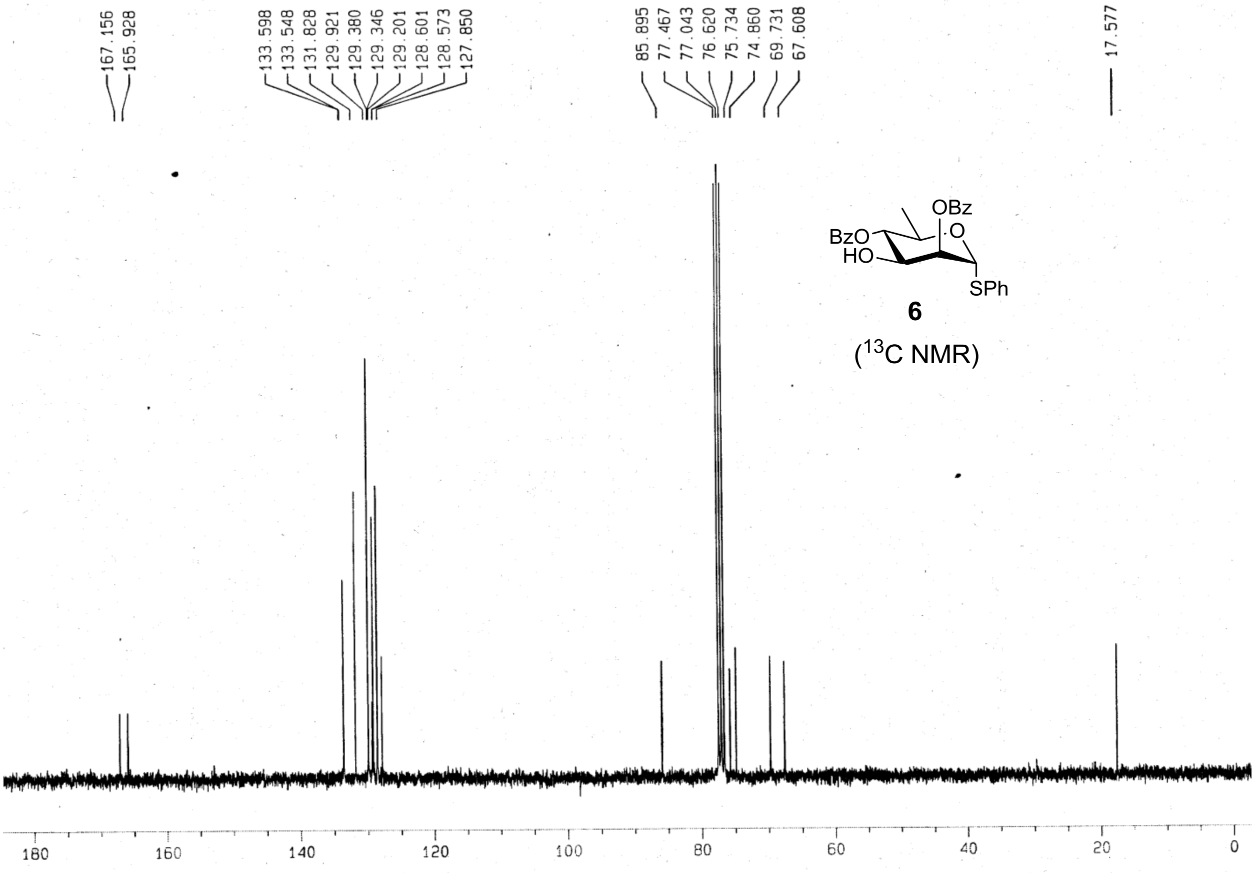
167.156
165.928
133.598
133.548
131.828
129.921
129.380
129.346
129.201
128.601
128.573
127.850
85.895
77.467
77.043
76.620
75.734
74.860
69.731
67.608
17.577
OBz
BzO
HO
O
SPh
6
(^{13}C NMR)
180
160
140
120
100
80
60
40
20
0

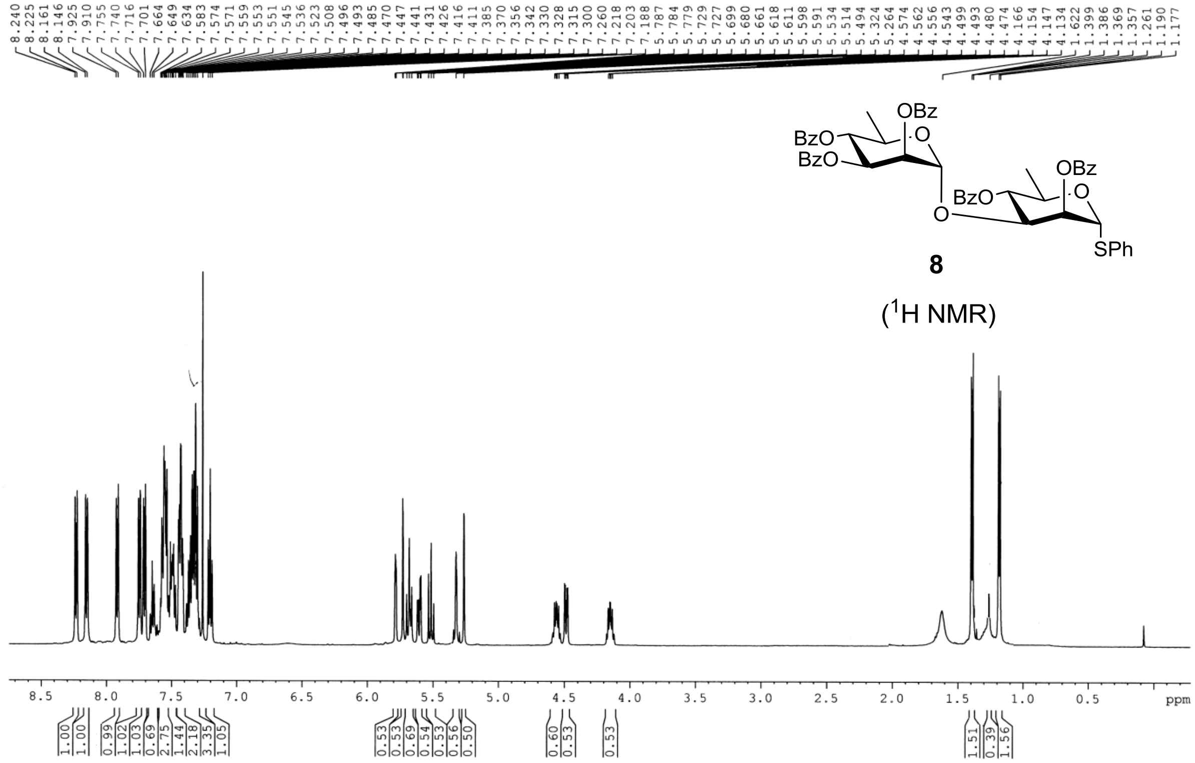
8.240 8.225 8.161 8.146 7.925 7.910 7.755 7.740 7.716 7.701 7.664 7.649 7.634 7.583 7.574 7.571 7.559 7.553 7.551 7.545 7.536 7.523 7.508 7.496 7.493 7.485 7.470 7.447 7.441 7.431 7.426 7.416 7.411 7.385 7.370 7.356 7.342 7.330 7.328 7.315 7.300 7.260 7.218 7.203 7.188 5.787 5.784 5.779 5.729 5.727 5.699 5.680 5.661 5.618 5.611 5.598 5.591 5.534 5.514 5.494 5.324 5.264 4.574 4.562 4.556 4.543 4.499 4.493 4.480 4.474 4.166 4.154 4.147 4.134 1.622 1.399 1.386 1.369 1.357 1.261 1.190 1.177
OBz
BzO
BzO
O
OBz
BzO
O
O
SPh
8
(1H NMR)
8.5 8.0 7.5 7.0 6.5 6.0 5.5 5.0 4.5 4.0 3.5 3.0 2.5 2.0 1.5 1.0 0.5 ppm
1.00 1.00 0.99 1.02 1.03 0.69 2.75 1.44 2.18 3.35 1.05 0.53 0.53 0.69 0.54 0.53 0.56 0.50 0.60 0.53 0.53 1.51 0.39 1.56

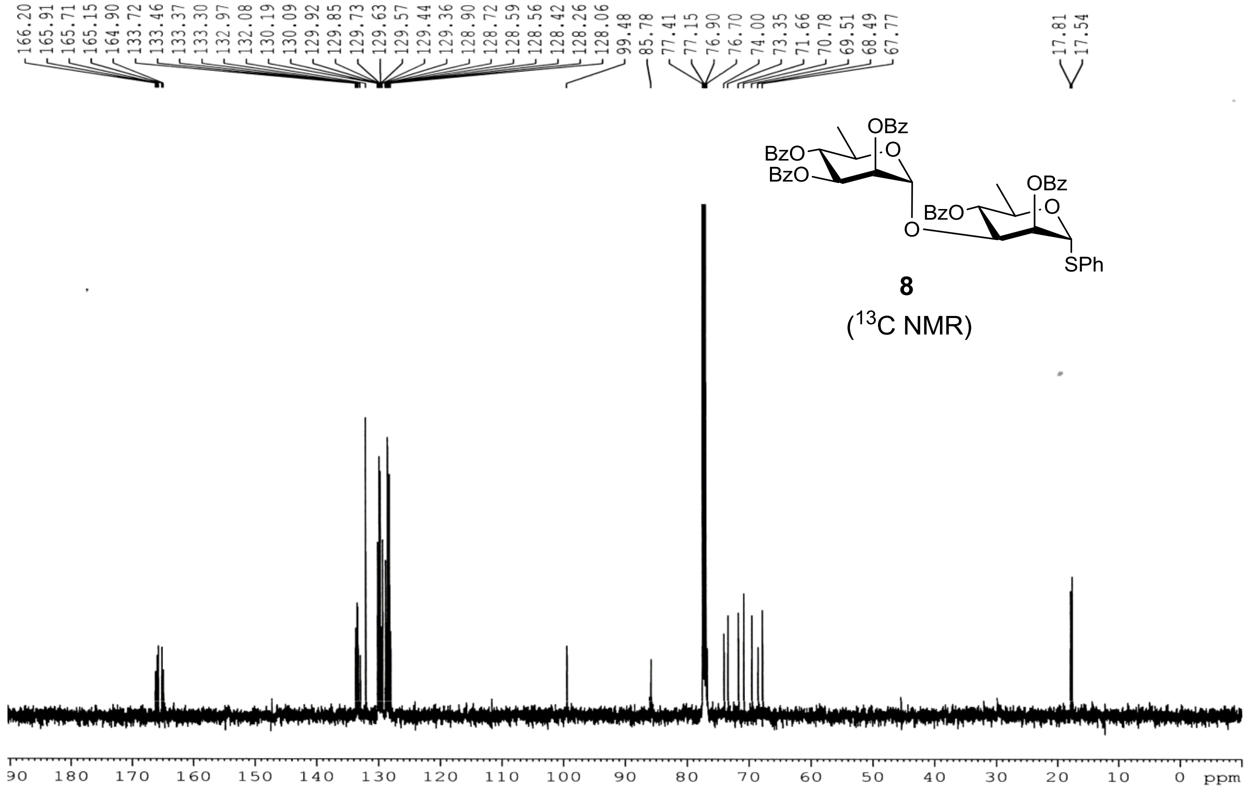
166.20 165.91 165.71 165.15 164.90 133.72 133.46 133.37 133.30 132.97 132.08 130.19 130.09 129.92 129.85 129.73 129.63 129.57 129.44 129.36 128.90 128.72 128.59 128.56 128.42 128.26 128.06 99.48 85.78 77.41 77.15 76.90 76.70 74.00 73.35 71.66 70.78 69.51 68.49 67.77 17.81 17.54
OBz
BzO
BzO
O
OBz
BzO
O
O
SPh
8
(13C NMR)
90 180 170 160 150 140 130 120 110 100 90 80 70 60 50 40 30 20 10 0 ppm

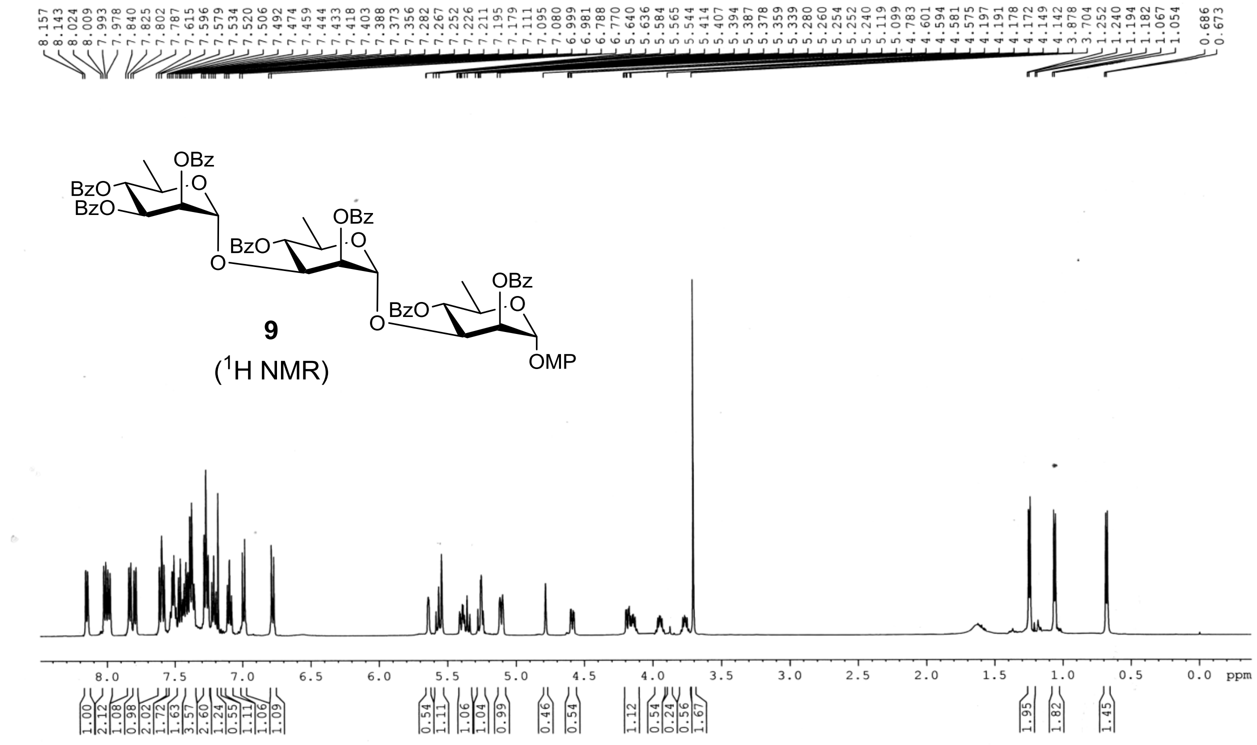
8.157
8.143
8.024
8.009
7.993
7.978
7.840
7.825
7.802
7.787
7.615
7.596
7.579
7.534
7.520
7.506
7.492
7.474
7.459
7.444
7.433
7.418
7.403
7.388
7.373
7.356
7.282
7.267
7.252
7.226
7.211
7.195
7.179
7.111
7.095
7.080
6.999
6.981
6.788
6.770
5.640
5.636
5.584
5.565
5.544
5.414
5.407
5.394
5.387
5.378
5.359
5.339
5.280
5.260
5.254
5.252
5.240
5.119
5.099
4.783
4.601
4.594
4.581
4.575
4.197
4.191
4.178
4.172
4.149
4.142
3.878
3.704
1.252
1.240
1.194
1.182
1.067
1.054
0.686
0.673
BzO
BzO
OBz
O
BzO
OBz
O
O
BzO
OBz
O
O
OMP
9
(1H NMR)
8.0
7.5
7.0
6.5
6.0
5.5
5.0
4.5
4.0
3.5
3.0
2.5
2.0
1.5
1.0
0.5
0.0
ppm
1.00
2.12
1.08
0.98
2.02
1.72
1.63
3.57
2.60
1.24
0.55
1.11
1.06
1.09
0.54
1.11
1.06
1.04
0.99
0.46
0.54
1.12
0.54
0.24
0.56
1.67
1.95
1.82
1.45

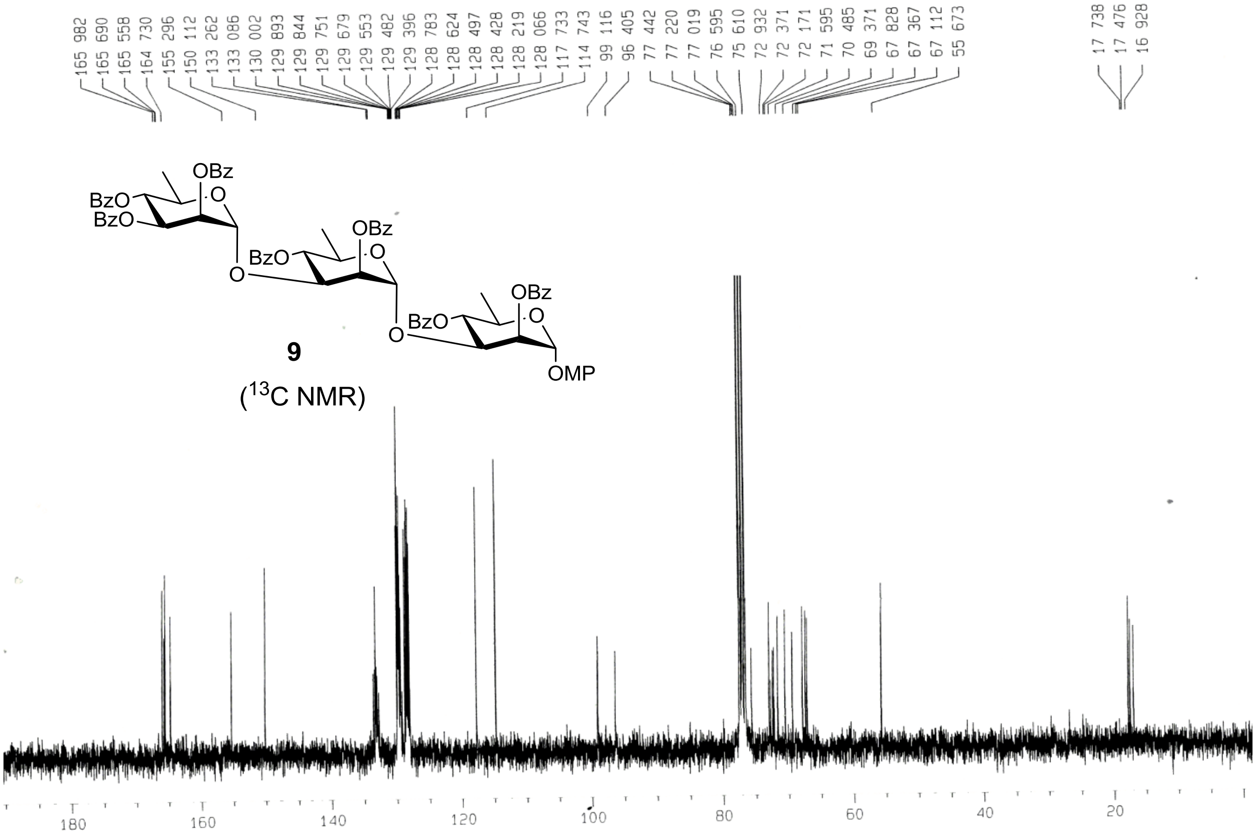
165 982
165 690
165 558
164 730
155 296
150 112
133 262
133 086
130 002
129 893
129 844
129 751
129 679
129 553
129 482
129 396
128 783
128 624
128 497
128 428
128 219
128 066
117 733
114 743
99 116
96 405
77 442
77 220
77 019
76 595
75 610
72 932
72 371
72 171
71 595
70 485
69 371
67 828
67 367
67 112
55 673
17 738
17 476
16 928
BzO
BzO
OBz
O
BzO
OBz
O
O
BzO
OBz
O
O
OMP
9
(13C NMR)
180
160
140
120
100
80
60
40
20

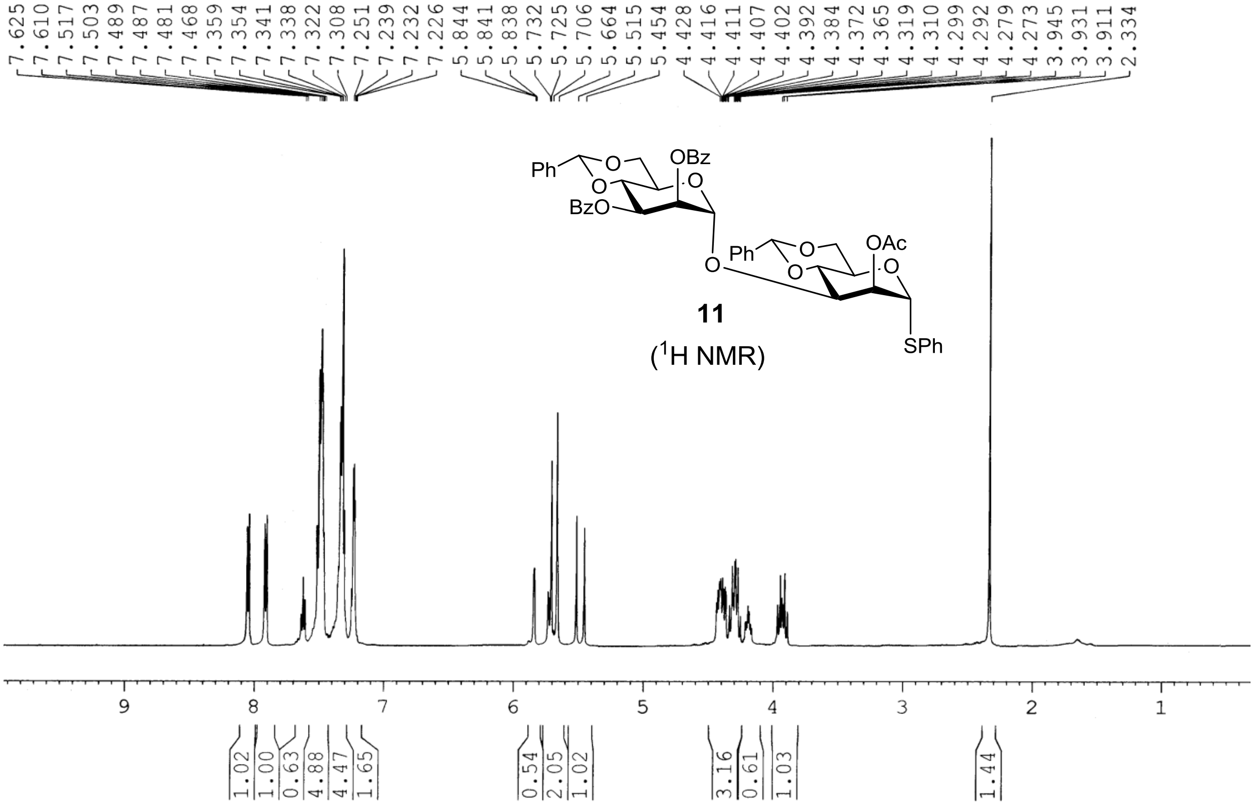
7.625 7.610 7.517 7.503 7.489 7.487 7.481 7.468 7.359 7.354 7.341 7.338 7.322 7.308 7.251 7.239 7.232 7.226 5.844 5.841 5.838 5.732 5.725 5.706 5.664 5.515 5.454 4.428 4.416 4.411 4.407 4.402 4.392 4.384 4.372 4.365 4.319 4.310 4.299 4.292 4.279 4.273 3.945 3.931 3.911 2.334
Ph
O
OBz
O
BzO
Ph
O
OAc
O
SPh
11
(¹H NMR)
1.02 1.00 0.63 4.88 4.47 1.65 0.54 2.05 1.02 3.16 0.61 1.03 1.44

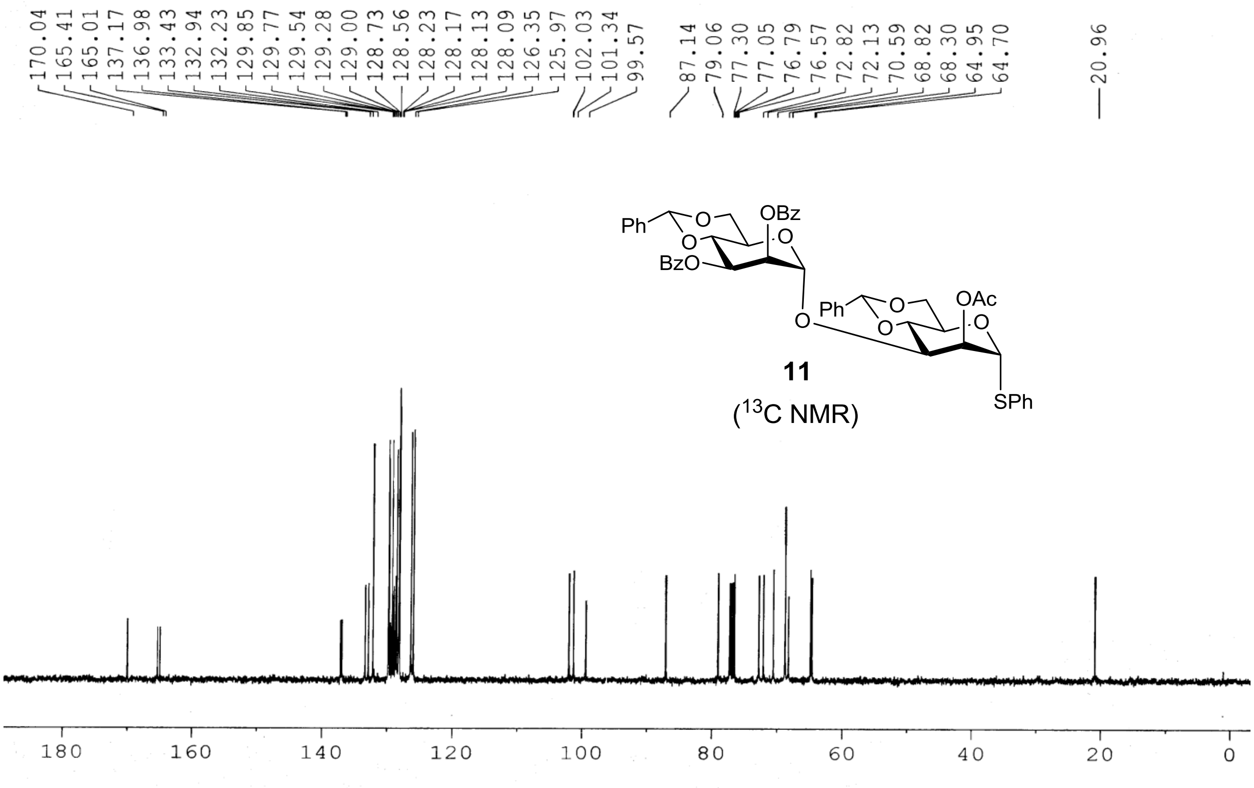
170.04 165.41 165.01 137.17 136.98 133.43 132.94 132.23 129.85 129.77 129.54 129.28 129.00 128.73 128.56 128.23 128.17 128.13 128.09 126.35 125.97 102.03 101.34 99.57 87.14 79.06 77.30 77.05 76.79 76.57 72.82 72.13 70.59 68.82 68.30 64.95 64.70 20.96
Ph
O
OBz
O
BzO
Ph
O
OAc
O
SPh
11
(¹³C NMR)

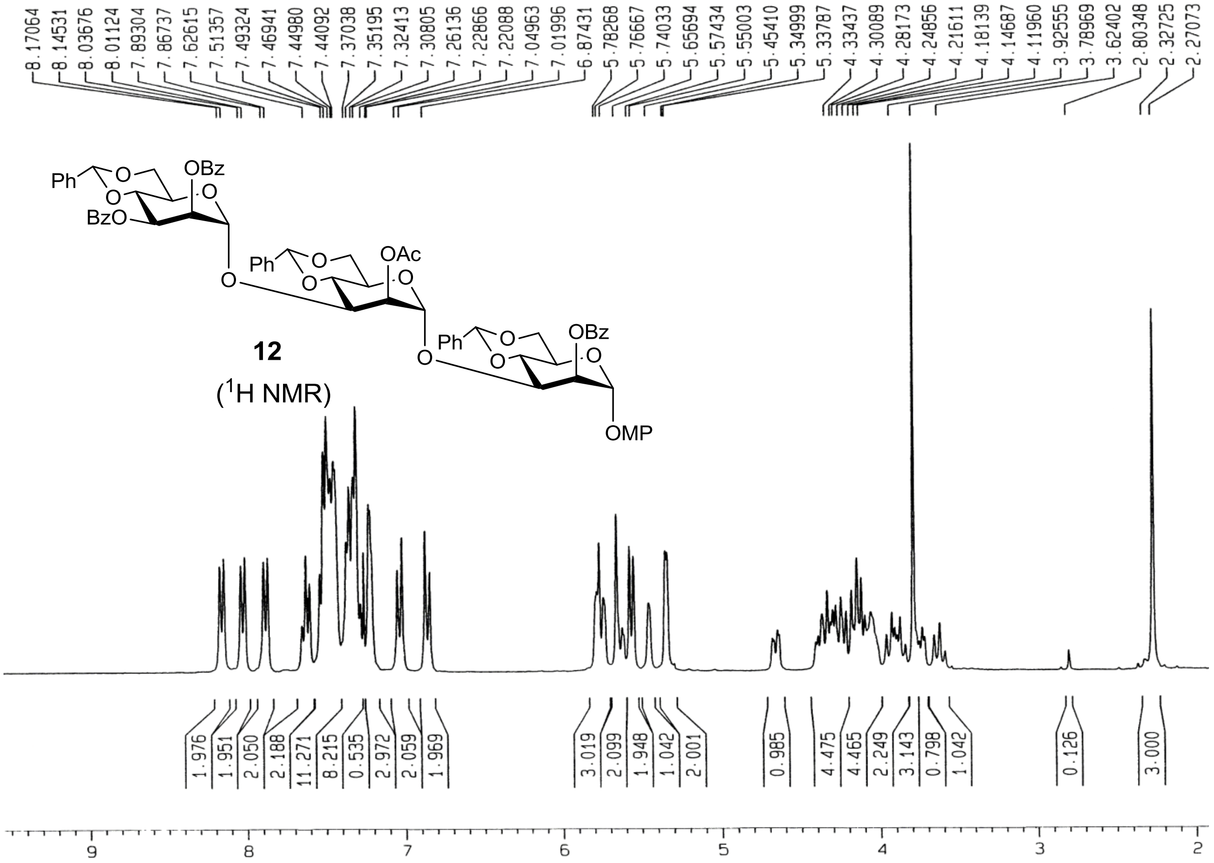
8.17064
8.14531
8.03676
8.01124
7.89304
7.86737
7.62615
7.51357
7.49324
7.46941
7.44980
7.44092
7.37038
7.35195
7.32413
7.30805
7.26136
7.22866
7.22088
7.04963
7.01996
6.87431
5.78268
5.76667
5.74033
5.65694
5.57434
5.55003
5.45410
5.34999
5.33787
4.33437
4.30089
4.28173
4.24856
4.21611
4.18139
4.14687
4.11960
3.92555
3.78969
3.62402
2.80348
2.32725
2.27073
Ph
OBz
BzO
OAc
OMP
12
(^{1}H NMR)
1.976
1.951
2.050
2.188
11.271
8.215
0.535
2.972
2.059
1.969
3.019
2.099
1.948
1.042
2.001
0.985
4.475
4.465
2.249
3.143
0.798
1.042
0.126
3.000
9
8
7
6
5
4
3
2
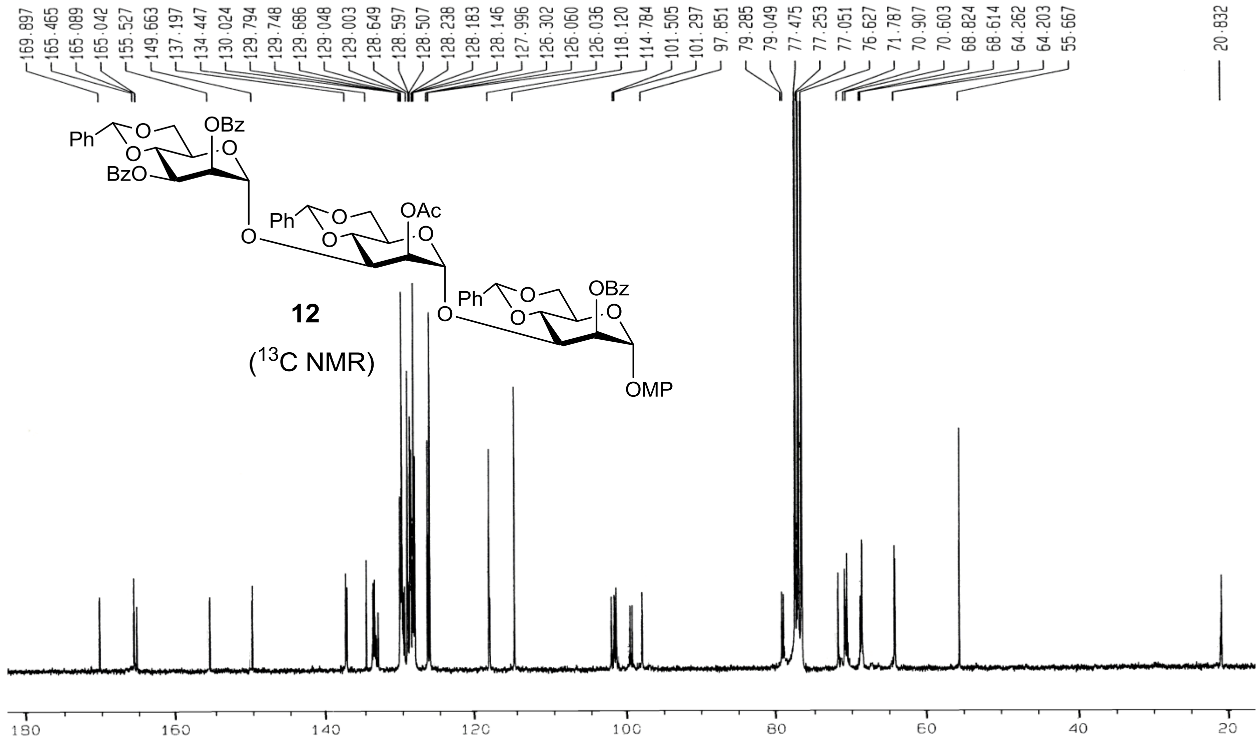
169.897
165.465
165.089
165.042
155.527
149.663
137.197
134.447
130.024
129.794
129.748
129.686
129.048
129.003
128.649
128.597
128.507
128.238
128.183
128.146
127.996
126.302
126.060
126.036
118.120
114.784
101.505
101.297
97.851
79.285
79.049
77.475
77.253
77.051
76.627
71.787
70.907
70.603
68.824
68.614
64.262
64.203
55.667
20.832
Ph
OBz
BzO
OAc
OMP
12
(^{13}C NMR)
180
160
140
120
100
80
60
40
20

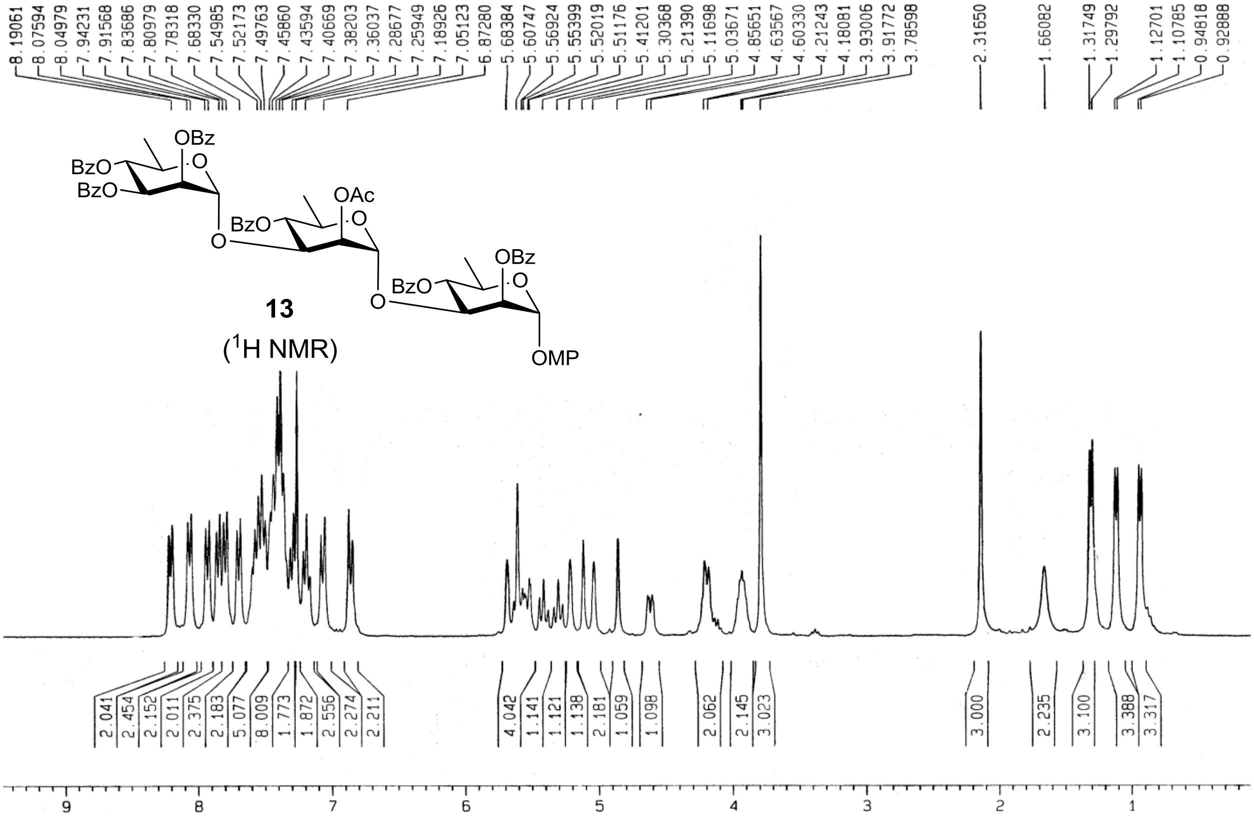
8.19061 8.07594 8.04979 7.94231 7.91568 7.83686 7.80979 7.78318 7.68330 7.54985 7.52173 7.49763 7.45860 7.43594 7.40669 7.38203 7.36037 7.28677 7.25949 7.18926 7.05123 6.87280
5.68384 5.60747 5.56924 5.55399 5.52019 5.51176 5.41201 5.30368 5.21390 5.11698 5.03671 4.85651 4.63567 4.60330 4.21243 4.18081 3.93006 3.91772 3.78598
2.31650
1.66082
1.31749 1.29792
1.12701 1.10785 0.94818 0.92888
BzO
BzO
OBz
O
BzO
OAc
O
O
BzO
OBz
O
O
OMP
13
(1H NMR)
2.041 2.454 2.152 2.011 2.375 2.183 5.077 8.009 1.773 1.872 2.556 2.274 2.211
4.042 1.141 1.121 1.138 2.181 1.059 1.098
2.062 2.145 3.023
3.000
2.235 3.100 3.388 3.317
9 8 7 6 5 4 3 2 1

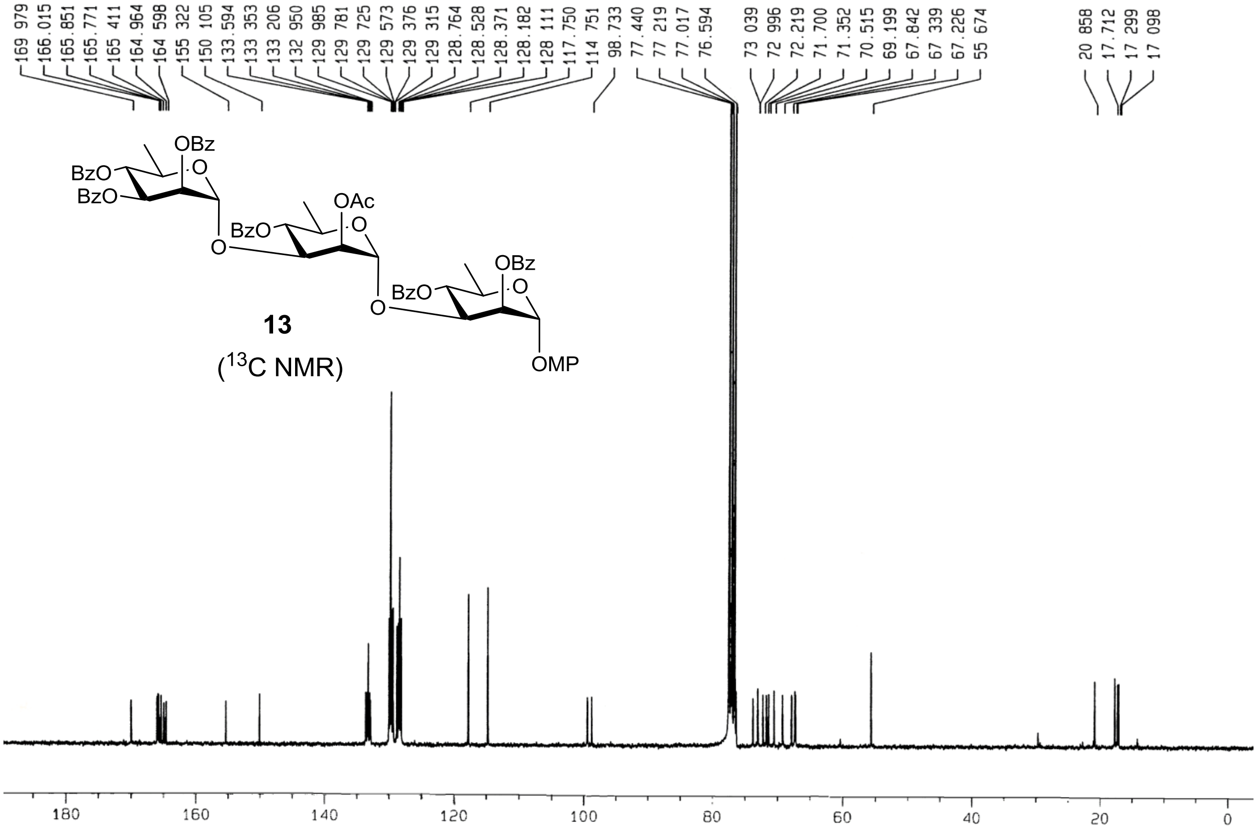
169 979 166.015 165.851 165.771 165 411 164.964 164 598 155 322 150 105 133.594 133 353 133 206 132 950 129 985 129 781 129 725 129 573 129 376 129 315 128.764 128.528 128.371 128.182 128 111 117.750 114 751 98.733
77.440 77 219 77.017 76.594
73 039 72 996 72.219 71.700 71.352 70.515 69.199 67.842 67 339 67.226 55 674
20 858 17.712 17 299 17 098
BzO
BzO
OBz
O
BzO
OAc
O
O
BzO
OBz
O
O
OMP
13
(13C NMR)
180 160 140 120 100 80 60 40 20 0

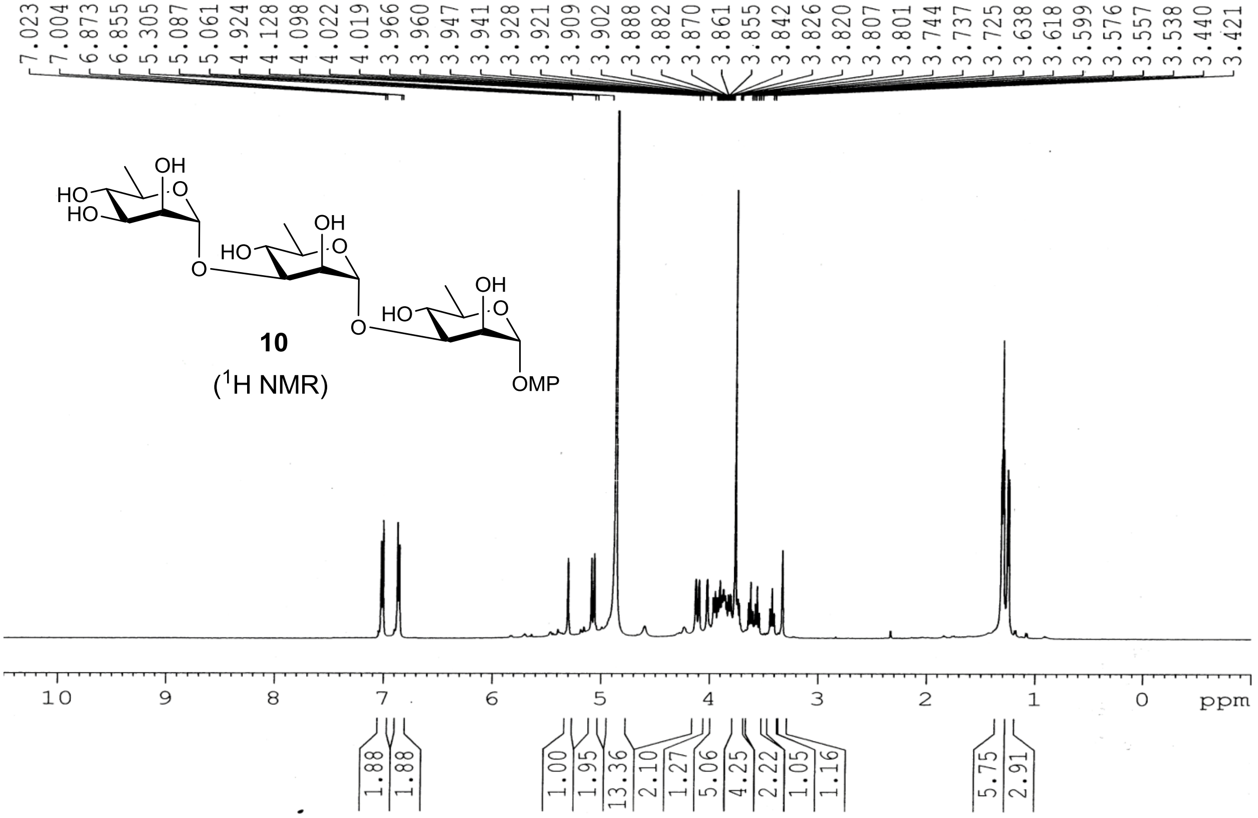
7.023 7.004 6.873 6.855 5.305 5.087 5.061 4.924 4.128 4.098 4.022 4.019 3.966 3.960 3.947 3.941 3.928 3.921 3.909 3.902 3.888 3.882 3.870 3.861 3.855 3.842 3.826 3.820 3.807 3.801 3.744 3.737 3.725 3.638 3.618 3.599 3.576 3.557 3.538 3.440 3.421
OH
HO
HO
O
OH
HO
O
OH
HO
O
OMP
10
(1H NMR)
10 9 8 7 6 5 4 3 2 1 0 ppm
1.88 1.88 1.00 1.95 13.36 2.10 1.27 5.06 4.25 2.22 1.05 1.16 5.75 2.91

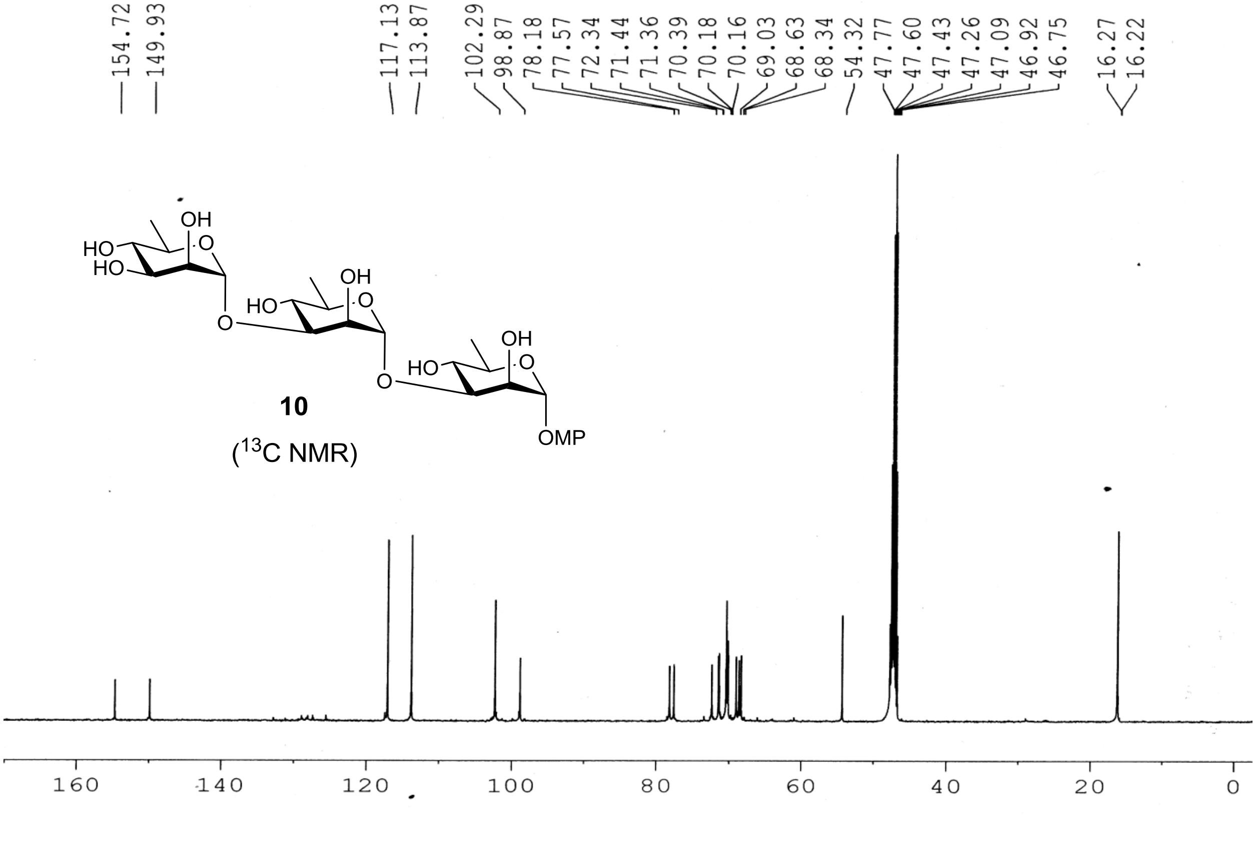
154.72 149.93 117.13 113.87 102.29 98.87 78.18 77.57 72.34 71.44 71.36 70.39 70.18 70.16 69.03 68.63 68.34 54.32 47.77 47.60 47.43 47.26 47.09 46.92 46.75 16.27 16.22
OH
HO
HO
O
OH
HO
O
OH
HO
O
OMP
10
(13C NMR)
160 140 120 100 80 60 40 20 0

^{1}H-^{1}H COSY spectra of compound 10

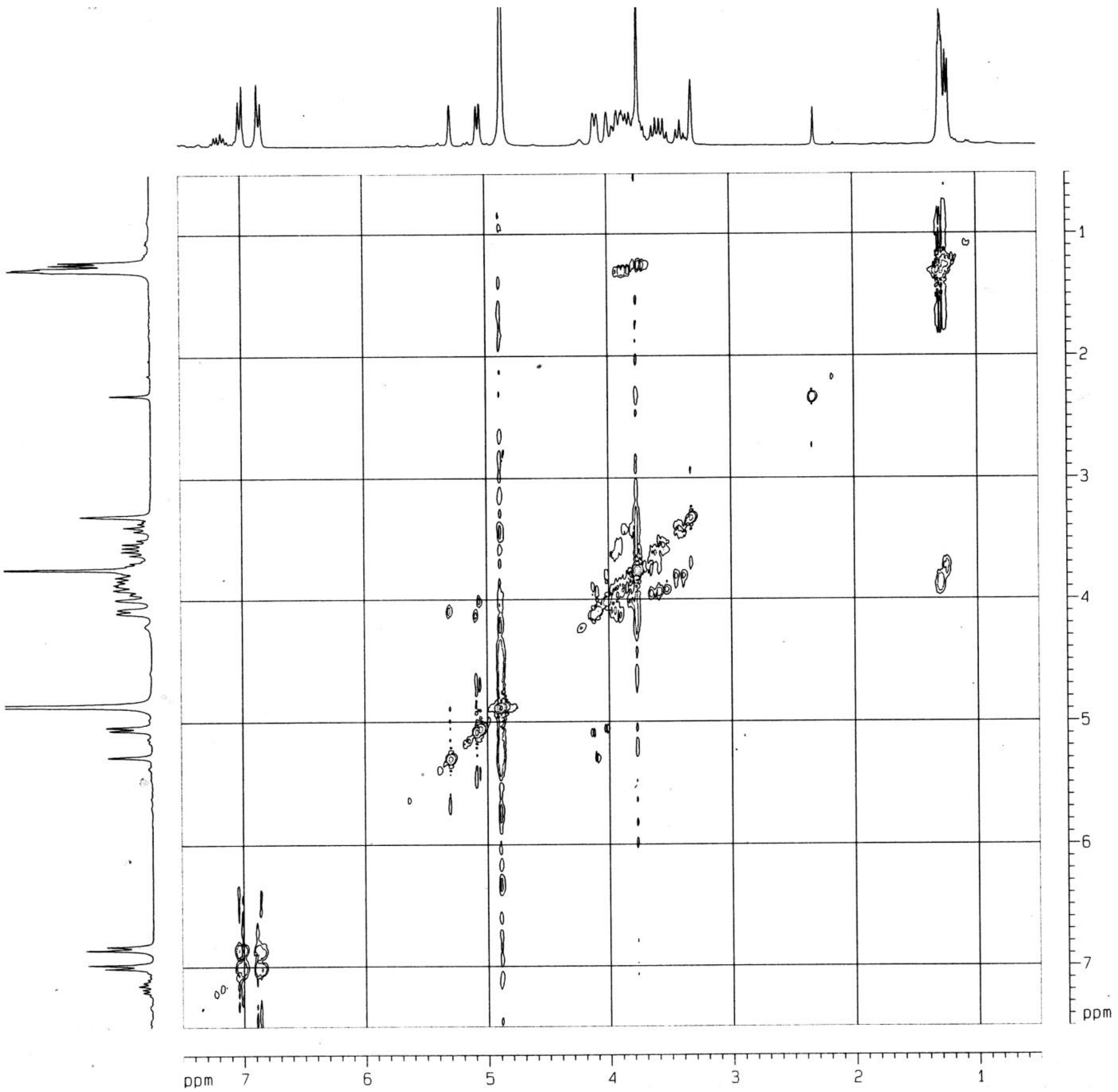

^{1}H-^{1}H COSY spectra contd.

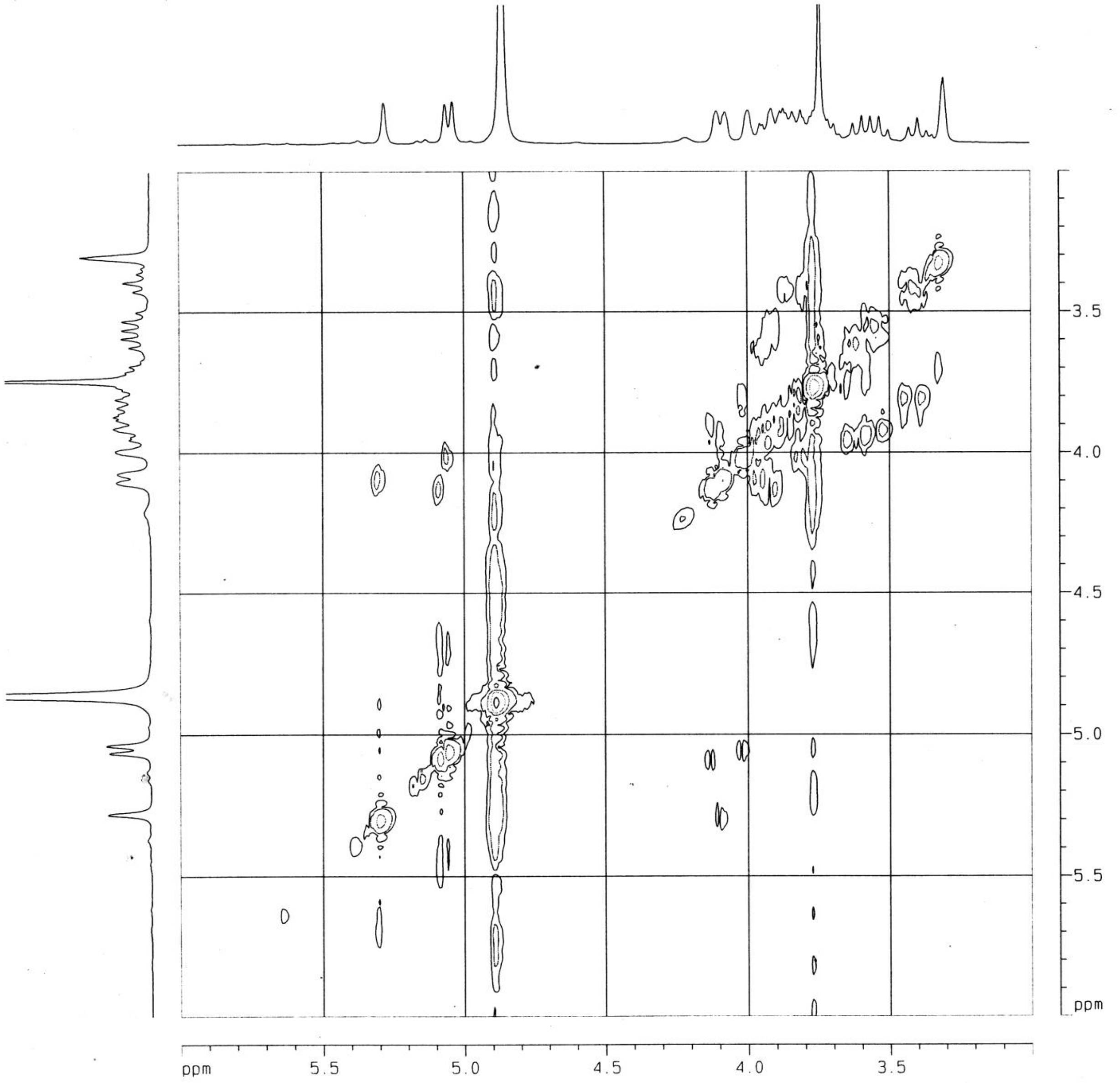

HSQC spectra of compound 10

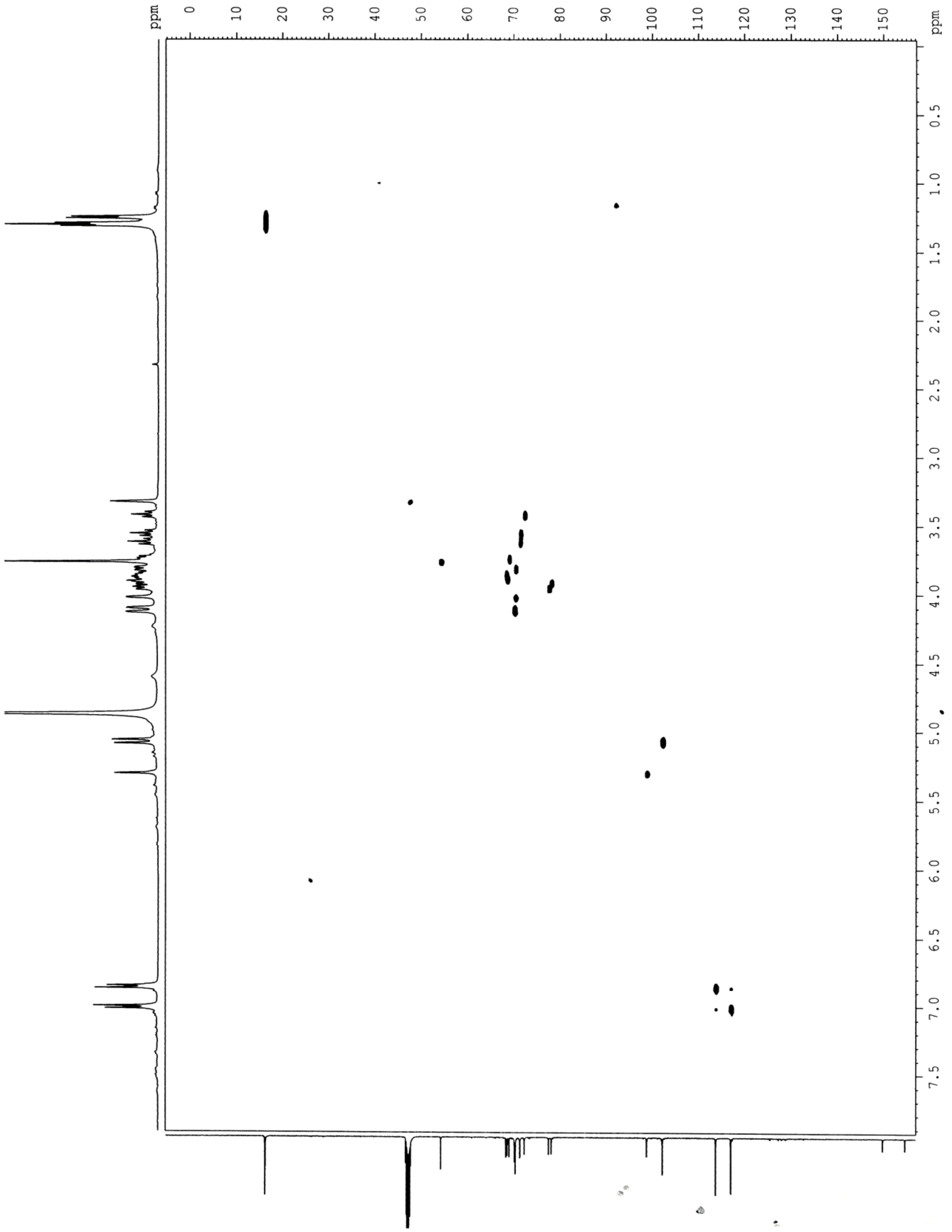

HSQC spectra contd.

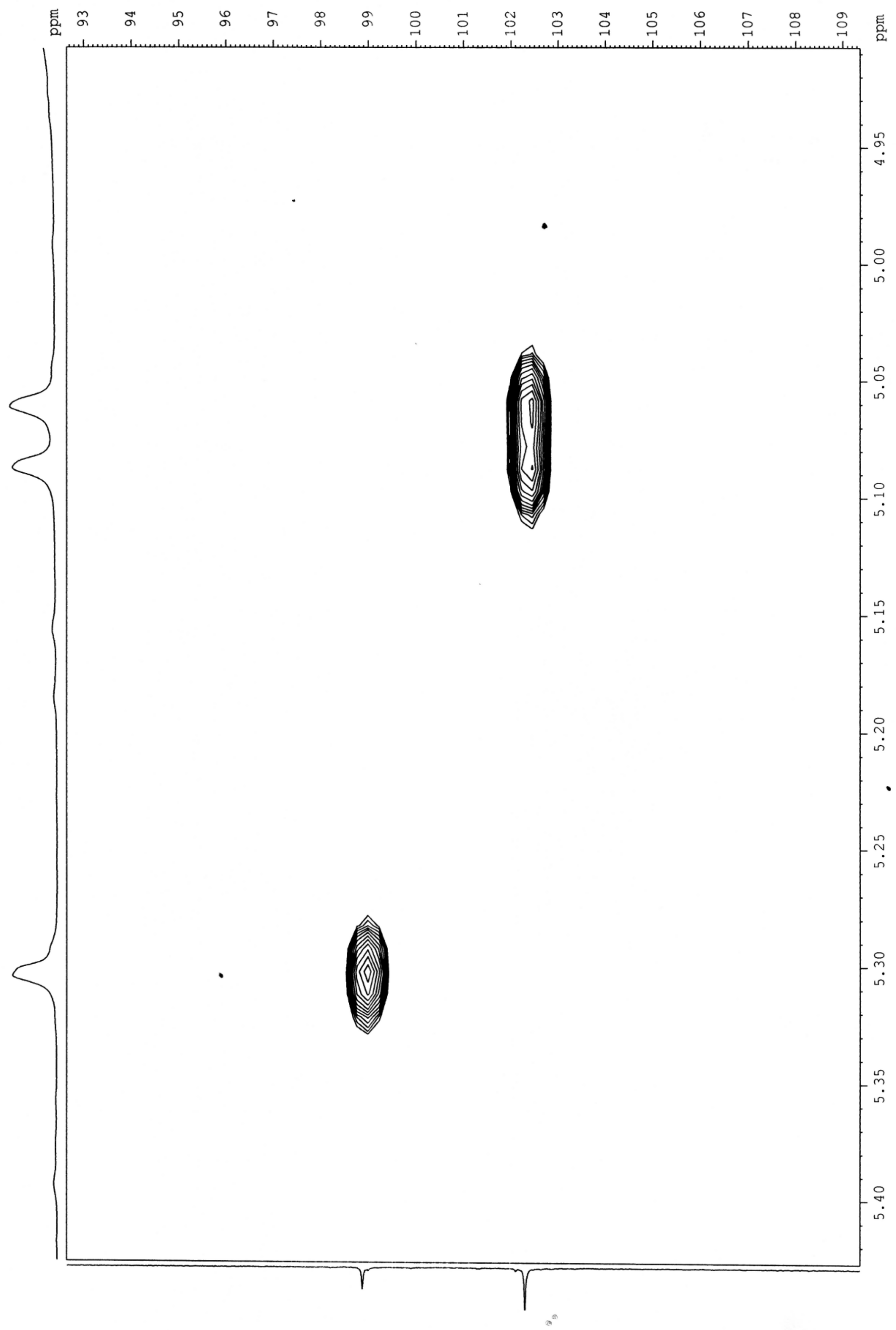

HSQC spectra contd.

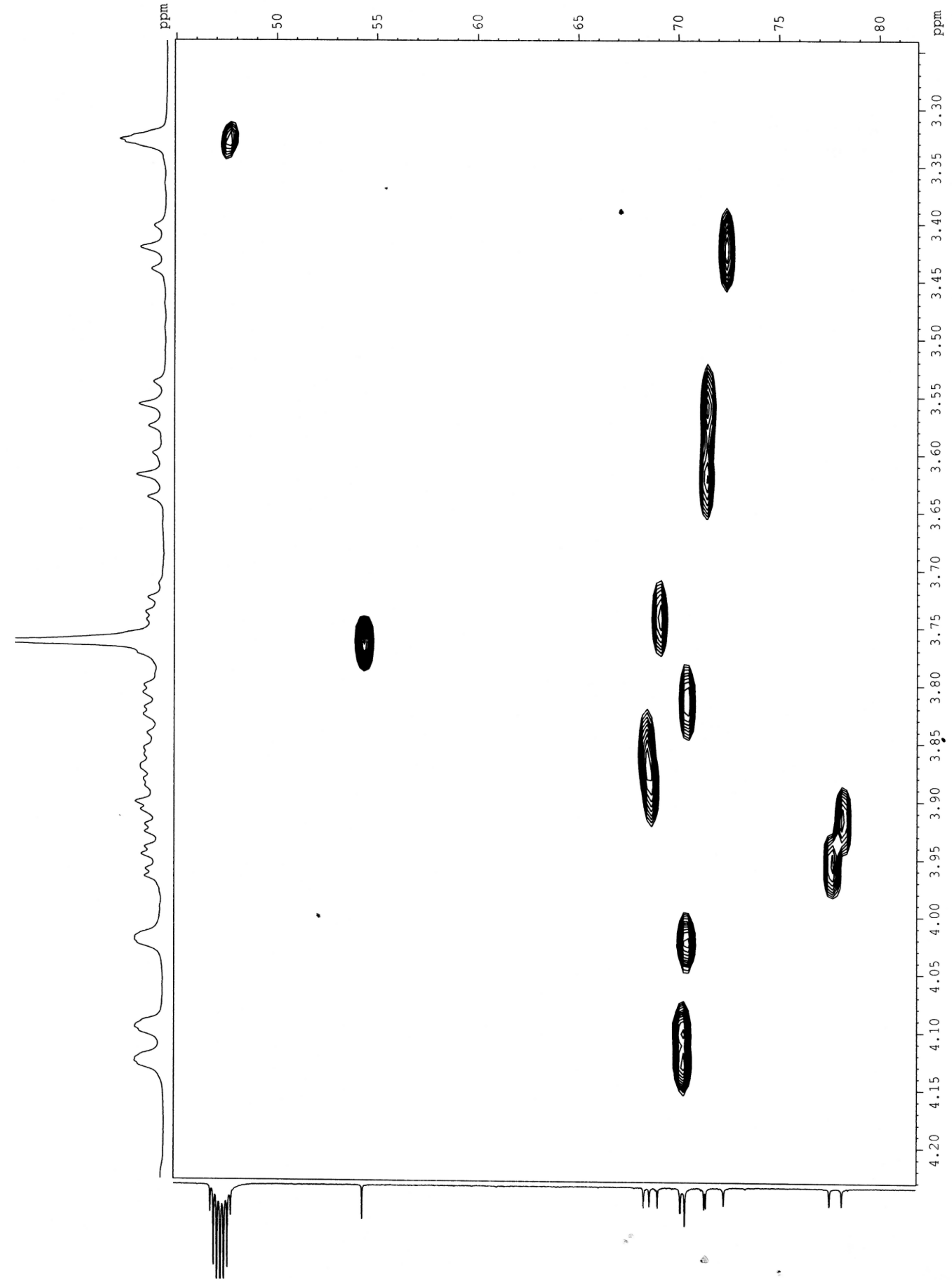

HMBC spectra of compound 10

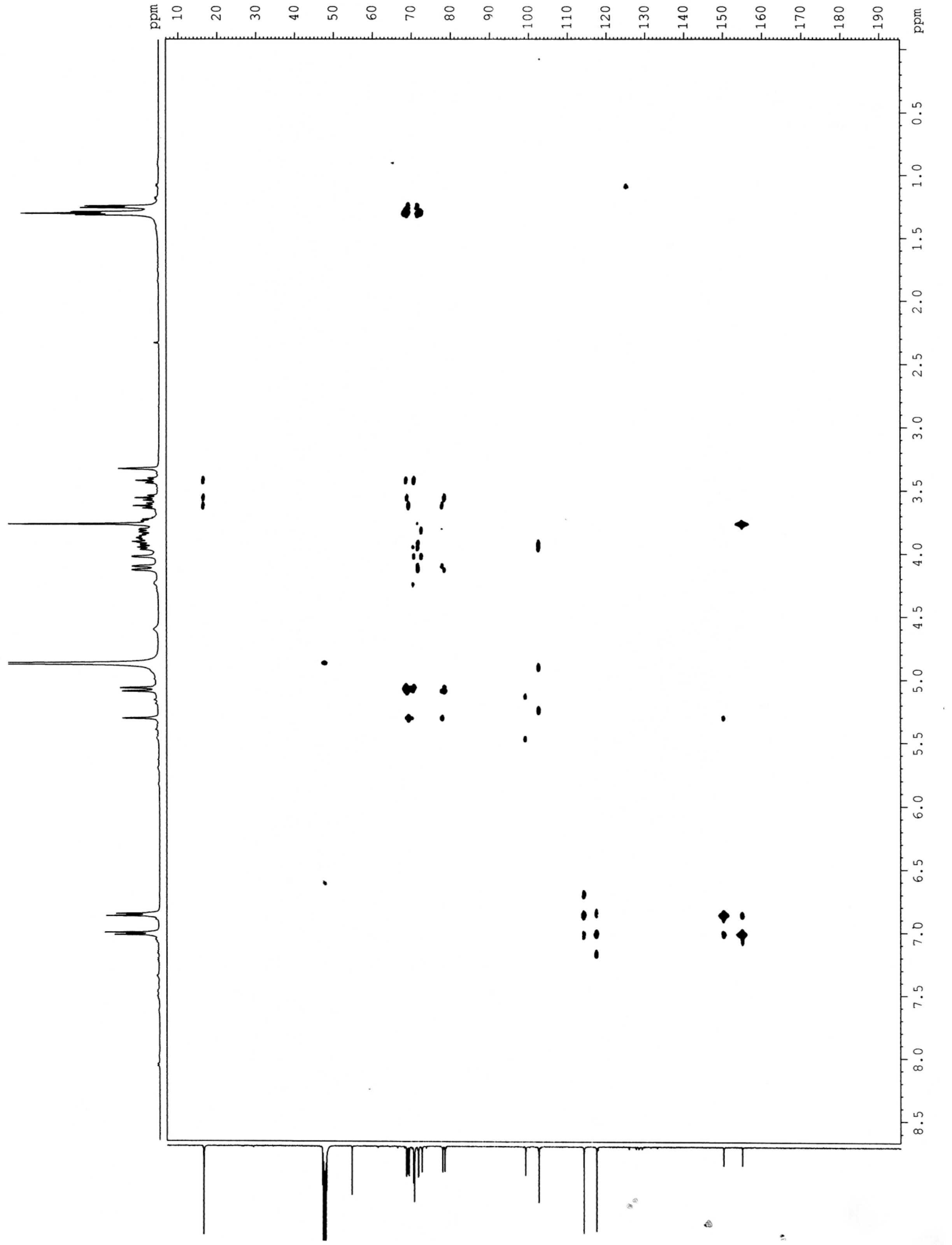

HMBC spectra contd.

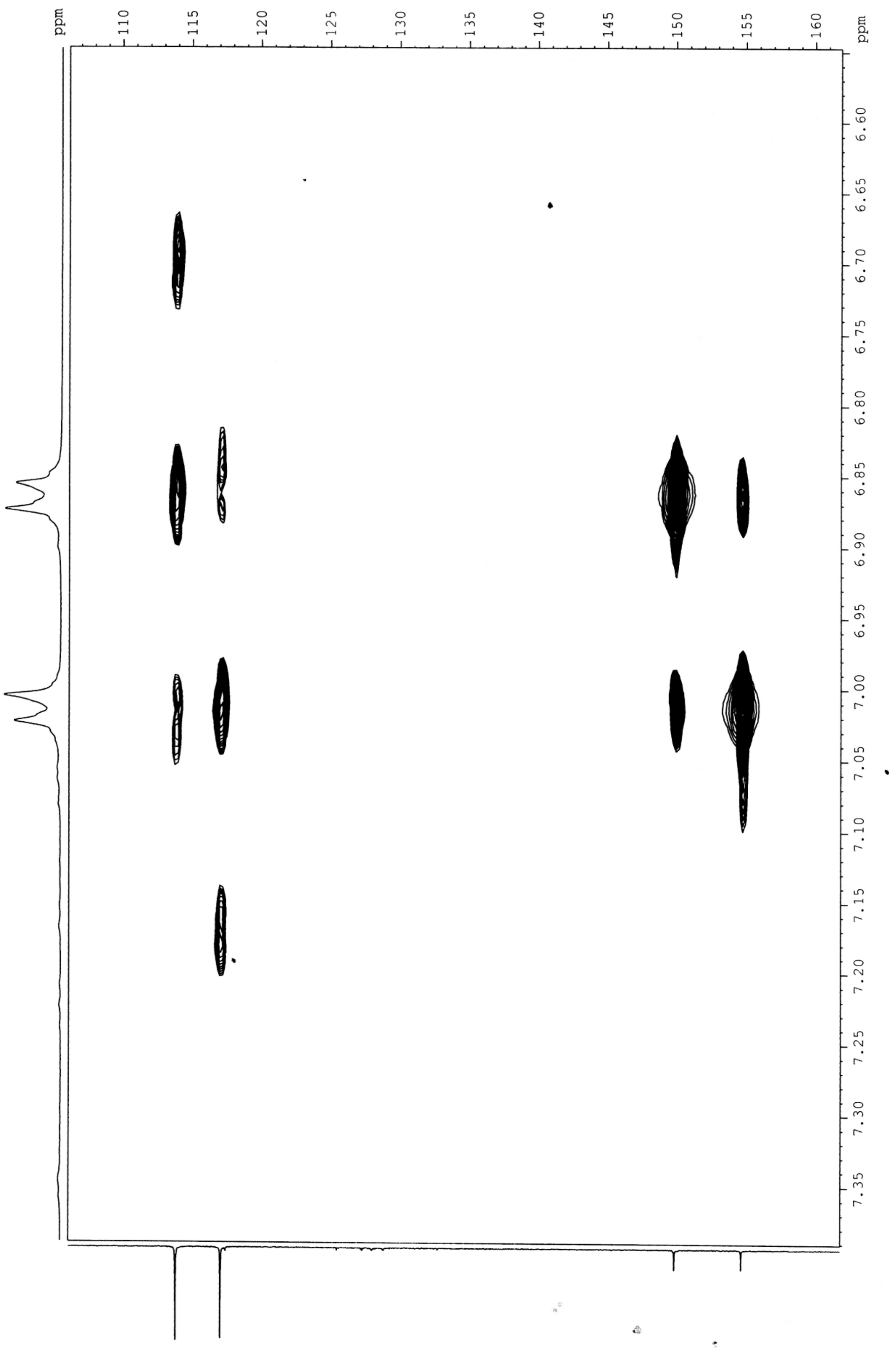

HMBC spectra contd.

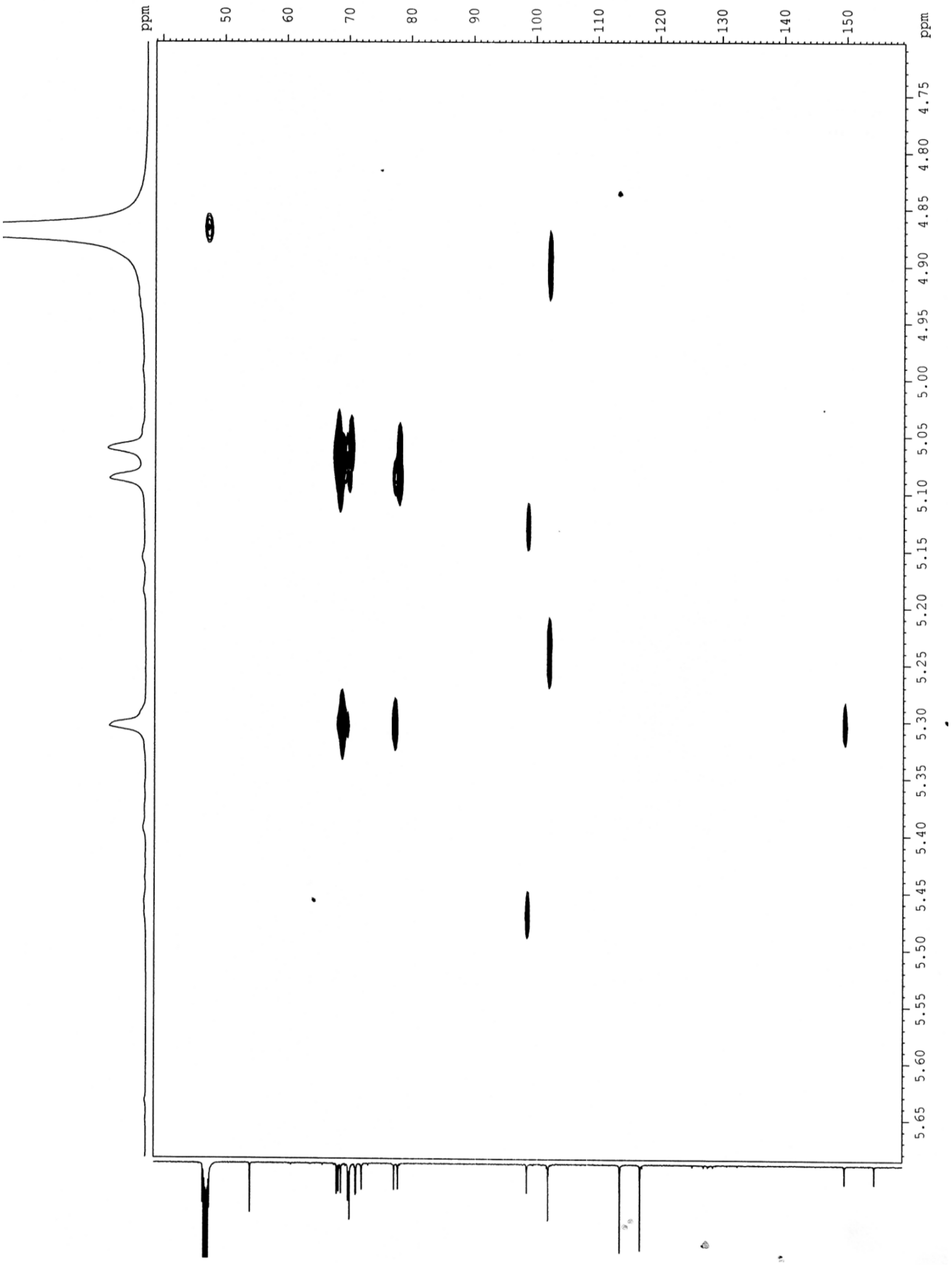

HMBC spectra contd.

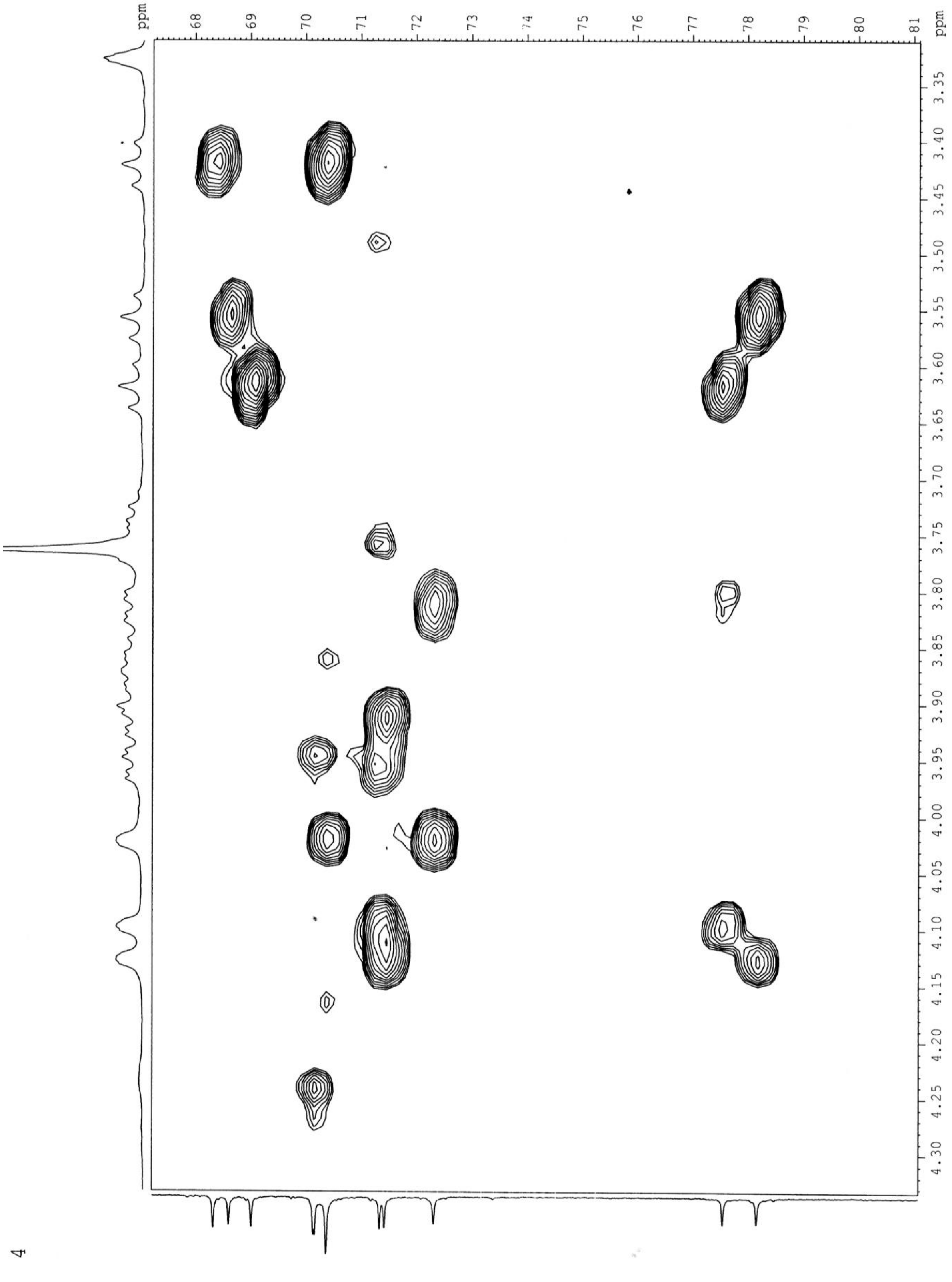

HMBC spectra contd.

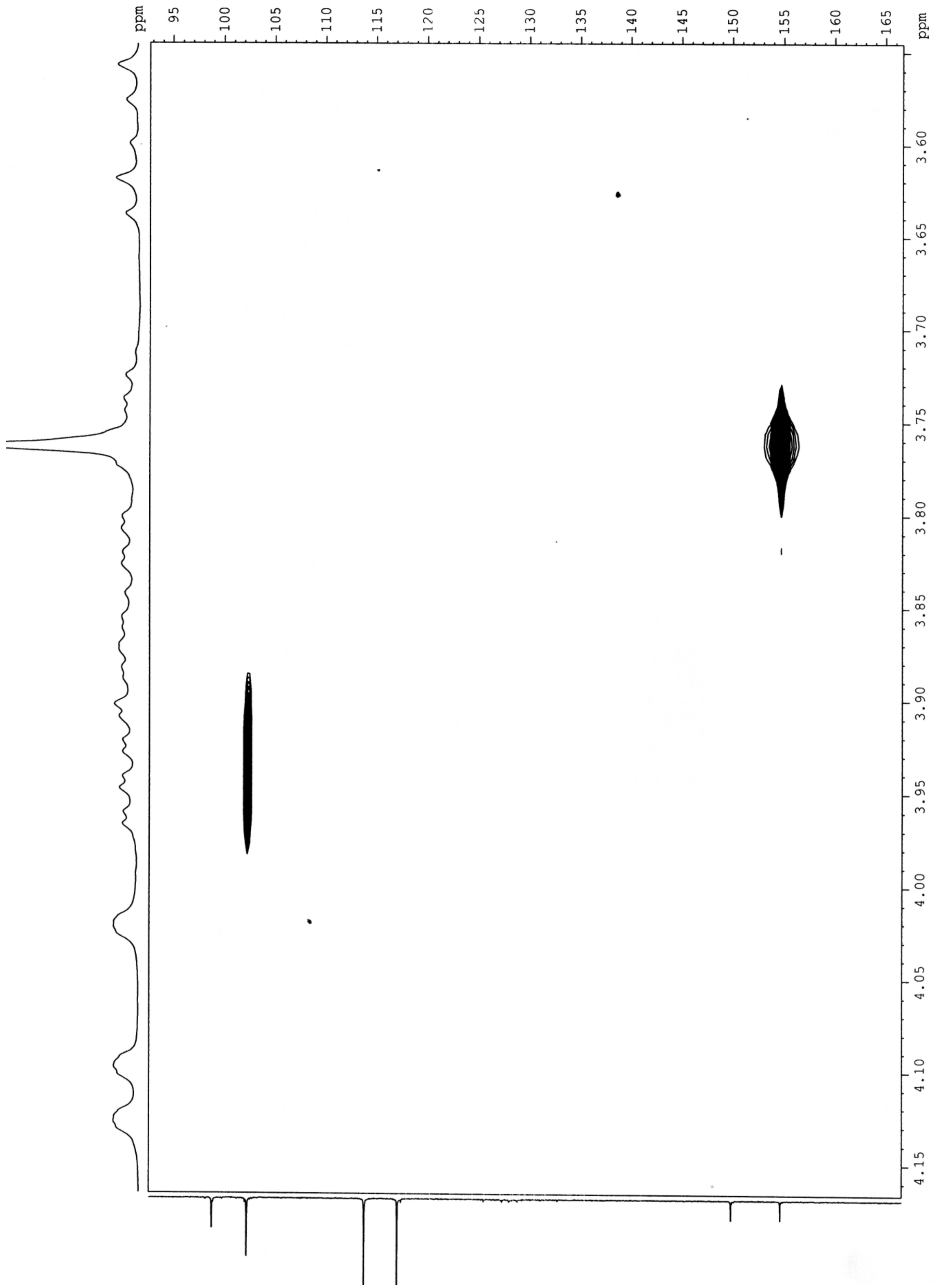

HMBC spectra contd.

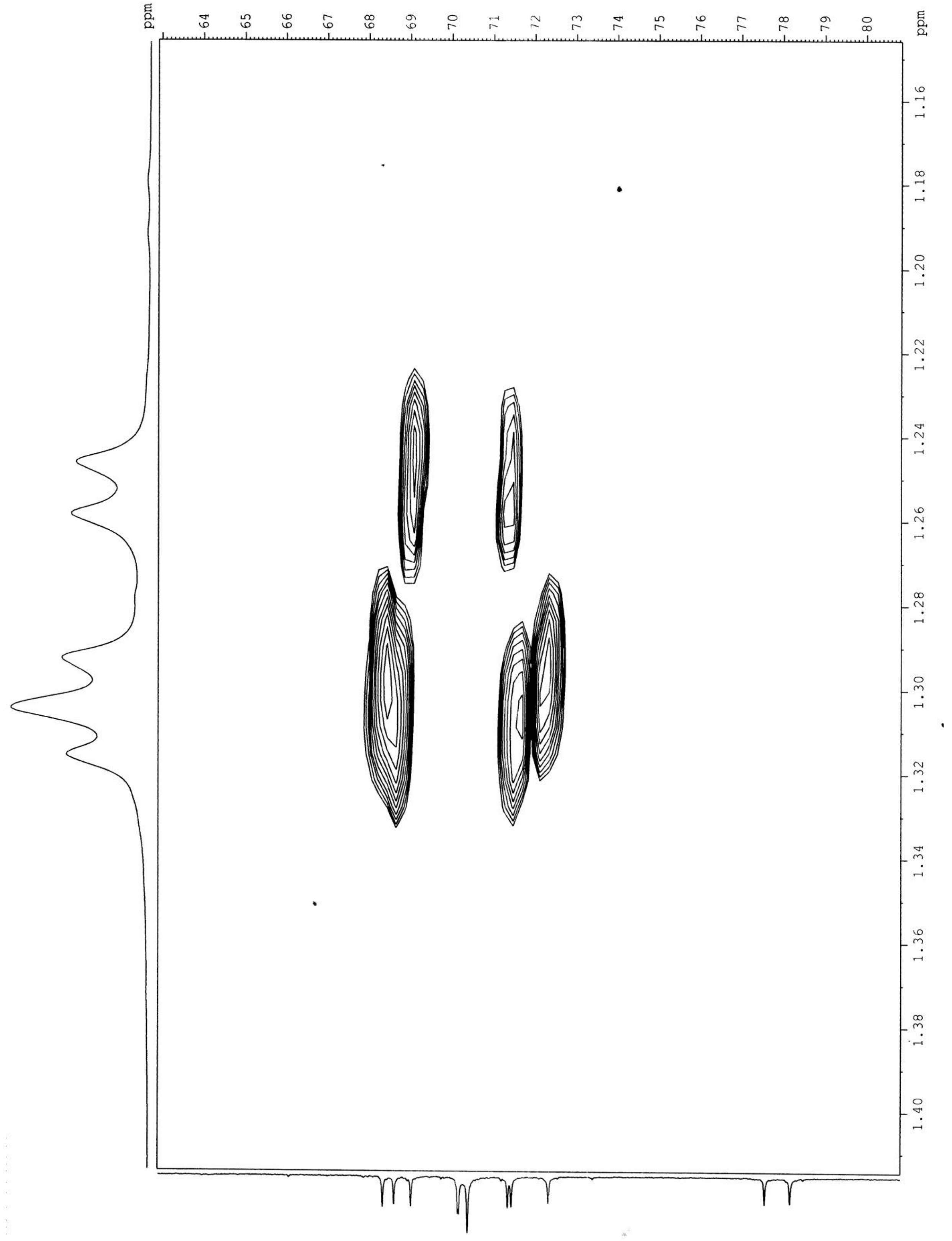

Supplement: File 2 — 1H and 13C NMR of compounds 2a, 4, 6, 8–13 and 2D NMR (COSY, HSQC and HMBC) of compound 10. [file Beilstein_J_Org_Chem-10-1488-s002.pdf]
